# The *Nicotiana tabacum* ABC transporter NtPDR3 secretes *O*-methylated coumarins in response to iron deficiency

**DOI:** 10.1093/jxb/ery221

**Published:** 2018-06-08

**Authors:** François Lefèvre, Justine Fourmeau, Mathieu Pottier, Amandine Baijot, Thomas Cornet, Javier Abadía, Ana Álvarez-Fernández, Marc Boutry

**Affiliations:** 1 Louvain Institute of Biomolecular Science and Technology, Université catholique de Louvain, Croix du Sud, Louvain-la-Neuve, Belgium; 2 Department of Plant Nutrition, Estación Experimental de Aula Dei, Consejo Superior de Investigaciones Científicas (CSIC), Zaragoza, Spain

**Keywords:** ABC transporter, coumarins, flavins, iron deficiency, *Nicotiana*, root exudates

## Abstract

Although iron is present in large amounts in the soil, its poor solubility means that plants have to use various strategies to facilitate its uptake. In this study, we show that expression of *NtPDR3*/*NtABCG3*, a *Nicotiana tabacum* plasma-membrane ABC transporter in the pleiotropic drug resistance (PDR) subfamily, is strongly induced in the root epidermis under iron deficiency conditions. Prevention of *NtPDR3* expression resulted in *N. tabacum* plants that were less tolerant to iron-deficient conditions, displaying stronger chlorosis and slower growth than those of the wild-type when not supplied with iron. Metabolic profiling of roots and root exudates revealed that, upon iron deficiency, secretion of catechol-bearing *O*-methylated coumarins such as fraxetin, hydroxyfraxetin, and methoxyfraxetin to the rhizosphere was compromised in *NtPDR3*-silenced plants. However, exudation of flavins such as riboflavin was not markedly affected by *NtPDR3*-silencing. Expression of *NtPDR3* in *N. tabacum* Bright Yellow-2 (BY-2) cells resulted in altered intra- and extracellular coumarin pools, supporting coumarin transport by this transporter. The results demonstrate that *N. tabacum* secretes both coumarins and flavins in response to iron deficiency and that *NtPDR3* plays an essential role in the plant response to iron deficiency by mediating secretion of *O*-methylated coumarins to the rhizosphere.

## Teaser

In response to iron deficiency, *Nicotiana tabacum* secretes both flavins and *O*-methylated coumarins to the rhizosphere, the latter being transported by NtPDR3, an ABC transporter.

## Introduction

Iron (Fe) is an essential micronutrient for all living organisms, including plants. For example, it is a component of heme and Fe-sulfur proteins involved in biological redox systems in the respiratory and photosynthetic electron transfer chains (cytochromes, ferredoxins, chlorophyll synthesis), and is also involved in stress responses (e.g. catalases and peroxidases), and in primary and secondary metabolism (e.g. cytochrome P450) (Hänsch and Mendel, 2009; Balk and Pilon, 2011). However, although Fe is the fourth most abundant element in the Earth’s crust, one third of the world’s cultivated soils are considered Fe-deficient. Iron has a strong tendency to form insoluble compounds, which makes it unavailable for uptake. Plants have therefore evolved various strategies to acquire Fe from the soil. When starved of Fe, non-grasses use a reduction-based strategy (Strategy I) for uptake. In contrast, grasses secrete Fe chelators and are able to acquire chelated Fe in the non-reduced form (Strategy II) (Marschner *et al.*, 1986).

Strategy I mainly relies on a three-step process, which consists of rhizosphere acidification and enhanced dissolution of Fe oxides, followed by the reduction of chelated Fe(III) to Fe(II) and the subsequent uptake of the ferrous ion. Several genes involved in Strategy I have been characterized. *FRO2* (ferric reductase oxidase 2) was shown to encode the main ferric chelate reductase in Arabidopsis roots (Yi and Guerinot, 1996; Robinson *et al.*, 1999). Subsequently, reduced Fe is taken up by roots via a transporter, known as IRT1 (iron-regulated transporter 1) in Arabidopsis (Henriques *et al.*, 2002; Varotto *et al.*, 2002; Vert *et al.*, 2002). Acidification possibly involves the plasma membrane H^+^-ATPase, and up-regulation of Arabidopsis H^+^-ATPase transcripts has been observed upon Fe deficiency (Santi and Schmidt, 2009). However, this has not been confirmed at the protein level (Lan *et al.*, 2011). In addition, it has been known for many years that Fe deficiency also stimulates the exudation of low molecular weight substances (Ambler *et al.*, 1971; Olsen *et al.*, 1981; Römheld and Marschner, 1983; Dakora and Phillips, 2002; Cesco *et al.*, 2010; Tsai and Schmidt, 2017). However, the role played by these compounds has long been underestimated owing to the minor involvement that they seem to have under conditions where Fe is readily available to the uptake machinery. Nevertheless, several reports have recently shown that Fe-deficient Arabidopsis plants up-regulate enzymes of the phenylpropanoid pathway, leading to the synthesis and secretion of phenolic compounds belonging to the coumarin family (Fourcroy *et al.*, 2014; Schmid *et al.*, 2014; Schmidt *et al.*, 2014; Sisó-Terraza *et al.*, 2016*a*; Ziegler *et al.*, 2016; Rajniak *et al.*, 2018; Tsai *et al.*, 2018). A transporter belonging to the ATP Binding Cassette (ABC) family, AtABCG37/AtPDR9, a member of the pleiotropic drug resistance (PDR) subfamily, has been suggested to mediate exudation of these compounds to the rhizosphere as *AtPDR9* was also shown to be up-regulated in response to Fe deficiency and plants silenced for *AtPDR9* expression failed to secrete coumarin derivatives out of the roots (Rodríguez-Celma *et al.*, 2013; Fourcroy *et al.*, 2014; Ziegler *et al.*, 2017). However, exudation of coumarins by Fe-depleted plants does not seem to be a universal rule. Indeed, upon Fe deficiency, *Medicago truncatula* specifically up-regulates genes involved in riboflavin biosynthesis and not those linked to the phenylpropanoid pathway (Rodríguez-Celma *et al.*, 2013). There is clearly a need for more examples before a generalized picture of the plant response to Fe deficiency can be drawn, and more direct evidence for the involvement of ABC transporters in coumarin transport is also needed.

*NtABCG3/NtPDR3*, the closest homolog of *AtPDR9* in *Nicotiana tabacum* (67% amino acid identity), has been shown to be expressed in *N. tabacum* suspension cells under conditions of Fe deficiency (Ducos *et al.*, 2005). However, *NtPDR3* expression and, in particular, its response to Fe deficiency have never been tested in the plant.

In this study, we show that the expression of *NtPDR3* is highly induced in the root epidermis upon Fe deficiency, and that preventing its expression results in increased sensitivity to low Fe supply. Characterization of the compounds accumulating in roots of Fe-deficient plants, as well as those secreted into the medium, showed that NtPDR3 is required for the export of *O*-methylated coumarins. Moreover, the results showed that the expression of *NtPDR3* in *N. tabacum* Bright Yellow-2 (BY-2) suspension cells modulated the intra- and extracellular pools of *O*-methylated coumarins. In addition to coumarins, flavins were also found in the roots and exudates of Fe-deficient *N. tabacum* plants, indicating that the secretion of coumarins and flavins are not always mutually exclusive. Overall, our data suggest that NtPDR3 plays an essential role in the plant response to Fe deficiency by exporting catechol-bearing *O*-methylated coumarins to the rhizosphere.

## Materials and methods

### Plant material and growth conditions

*Nicotiana tabacum* cv Petit Havana SR1 (Maliga *et al.*, 1973) was used as the wild-type. For *in vitro* culture, plants were grown at 24 °C on solid Hoagland medium [5 mM Ca(NO_3_)_2_, 5 mM MgSO_4_, 5 mM KNO_3_, 1 mM KH_2_PO_4_, 10 µM Fe(III)-EDTA, 5 µM H_3_BO_3_, 4.5 µM MnCl_2_, 3.8 µM ZnSO_4_, 0.1 µM (NH_4_)_6_Mo_7_O_24_, 0.3 µM CuSO_4_, 0.6% (w/v) agar, pH 5.8]. Kanamycin (100 mg l^–1^) was used to select *N. tabacum* transgenic lines. The photoperiod was 16 h per day with a photosynthetic photon flux density (PPFD) of 50 µmol photons m^–2^ s^–1^. For hydroponic culture, *N. tabacum* plants were grown in 4× diluted Hoagland medium (five plants in 2 l of solution) under a day/night regime of 16/8 h (25/18 °C) with a PPFD of 250 µmol photons m^–2^ s^–1^. The nutrient solution was renewed every week. Hydroponic cultures were started from 4-week-old *in vitro*-cultured plants. All experiments involving hydroponically grown plants were performed after 2 weeks under hydroponic conditions.

### Plant transformation

For the *GUS* (*β-glucuronidase*) construct, a 1502-bp sequence upstream of the *NtPDR3* ATG initiating codon was amplified (5′-aaactgcagcgatttgattggctatggaac-3′ and 5′-cgcggatcccaactgagccatttttggtg-3′) and fused to the *GUS* reporter gene, previously inserted (*EcoRI*/*HindIII*) into the pAUX3131 vector (Goderis *et al.*, 2002). The *pNtPDR3::GUS* expression cassette was then transferred into the *I-SceI* site of the pPZP-RCS2 binary vector (Goderis *et al.*, 2002) containing a neomycin phosphotransferase expression cassette.

For the artificial miRNA (amiRNA)-silencing construct, the amiRNA TATCCGTTGATATAATGGGGC sequence was engineered into the pRS300 plasmid by site-directed mutagenesis following the instructions for Web MicroRNA Designer (http://wmd3.weigelworld.org; Ossowski *et al.*, 2008) and using the primers 5′-gaTATCCGTTGATATAA TGGGGCtctctcttttgtattcc-3′ (primer I), 5′-gaGCCCCATTATATCAA CGGATAtcaaagagaatcaatga-3′ (primer II), 5′-gaGCACCATTATATCTA CGGATTtcacaggtcgtgatatg-3′ (primer III), and 5′-gaAATCCGTAGATATAA TGGTGCtctacatatatattcct-3′ (primer IV). The amiRNA foldback was introduced (*KpnI*/*SacI*) into the pAUX3131-En2pPMA4-tNOS plasmid (De Muynck *et al.*, 2009), and then the amiRNA expression cassette was transferred into the *I-SceI* site of the pPZP-RCS2 binary vector (Goderis *et al.*, 2002).

The binary vectors were transferred into *Agrobacterium tumefaciens* LBA4404 virGN54D (van der Fits *et al.*, 2000) for stable transformation of *N. tabacum* cv Petit Havana SR1, as described previously (Horsch *et al.*, 1985). Transformed plants were selected on solid medium [0.44% (w/v) Murashige and Skoog (MS) salts (ICN Biomedicals), pH 5.6, 3% (w/v) sucrose, 1% (w/v) agar] supplemented with 100 mg l^–1^ of kanamycin and grown at 24 °C. The photoperiod was 16 h per day with a PPFD of 50 µmol photons m^–2^ s^–1^.

### Metabolic profiling

*Nicotiana tabacum* plants grown for 2 weeks in hydroponic culture were transferred to a new medium supplemented with or without 10 µM Fe(III)-EDTA. Then, at 3 d after transfer to the new medium, the nutrient solutions were collected and filtered using 0.45-µm PVDF filters. Metabolites were then retained on SepPak C18 cartridges (Waters), eluted with 2 ml of methanol, and stored at –80 °C until further analysis. In parallel, roots were frozen in liquid nitrogen and ground for 3 min (Retsch M301 ball mill). Samples of 100 mg (FW) were then homogenized twice in 1 ml of methanol for 5 min. Supernatants were pooled, vacuum dried, and dissolved with 250 µl methanol.

HPLC/DAD-ESI-MS (time-of flight, TOF) and HPLC/ESI-MS/MS (ion trap) analyses were carried out as described by Sisó-Terraza *et al.* (2016*a*). Molecular formulae were assigned based on the exact molecular weight with errors <5 ppm.

Quantification of coumarins and their glycosides was carried out using [M+H]^+^ signals in the HPLC/ESI-MS (TOF). Internal standards (IS) were added in the root and nutrient extracts, and the occurrence of isobaric compounds that might have co-eluted with them had previously been checked in order to avoid analytical interferences in the quantification process. Extractions were carried out with addition of the following IS compounds: artemicapin C, a methylenedioxy-coumarin, for quantification of the coumarins scopoletin, fraxetin isomer, methoxyfraxetin, isofraxidin and fraxinol; esculetin, for quantification of oxidized hydroxyfraxetin, hydroxyfraxetin, and fraxetin, and the glucoside form of the coumarin esculetin, for quantification of coumarin glycosides. ISs were added to the extracts at the following concentrations: 5 µM artemicapin C, 10 µM esculetin, and 20 µM esculin. Quantification of fraxetin, scopoletin, isofraxidin, fraxinol, and scopolin was carried out using authenticated standards. For other coumarins, as no commercial standards are available, concentrations were estimated as fraxetin for free coumarins, and as fraxin for glycosides.

Quantification of flavins was performed directly by using the absorption peak area at 445 nm using their authenticated standards. As no commercial standard was available for riboflavanal, the concentration of this compound was estimated as riboflavin.

### Construction of the *pHSP3A::10His/STREPII/TEV-NtPDR3* expression vector

The *NtPDR3* coding sequence was provided with an N-terminal double-purification tag, 10-His and STREPII, followed by the tobacco etch virus (TEV) protease cleavage site (see Supplementary Fig. S1 at *JXB* online for sequence). The 187-bp sequence corresponding to the tag flanked by the KpnI and BglII restriction sites was ordered through the GeneArt^©^ Strings^TM^ facility (ThermoFisher Scientific). This fragment was then inserted in the pAUX3131 auxiliary plasmid between the tNOS terminator and the heat-inducible pHSPA3A transcriptional promoter (Navarre *et al.*, 2011). The *pHSP3A::10His/STREPII/TEV-NtPDR3* expression cassette was released from this plasmid by I-SceI and inserted in the pPZP- RCS2 binary vector (Goderis *et al.*, 2002).

### Transformation of *N. tabacum* BY-2 cells

Transgenic lines of *N. tabacum* BY-2 were obtained as described by Piette *et al.* (2011).

### GUS histochemical analysis

GUS activity in *N. tabacum* plants was measured as described previously by Bultreys *et al.* (2009).

### RNA preparation and quantitative RT-PCR

Plant tissues (100 mg, FW) were ground in liquid nitrogen using a mortar and pestle. RNA extraction was performed using the Spectrum^TM^ Plant Total RNA Kit (Sigma-Aldrich, http://www.sigmaaldrich.com) according to the manufacturer’s specifications. Genomic DNA was eliminated by using the On-Column DNase I Digestion Set (Sigma-Aldrich) on a spin column. DNA-free RNA (2 µg) was used for reverse transcription using the Moloney Murine Leukemia Virus Reverse transcriptase (Promega, https://be.promega.com) and oligo(dT)_18_. Samples were incubated for 5 min at 25 °C, followed by 1 h at 42 °C and 5 min at 85 °C. Gene-specific PCR primers (about 100 bp; melting temperature, 60 °C) were designed in the 3′-end of the coding sequence using OligoPerfect™ Designer (http://www.thermofisher.com); primers are listed in Supplementary Table S1. We used 5 µl of 10× diluted cDNAs as a template in a 20-µl RT-qPCR reaction, which also contained 10 µl of the Power SYBR green PCR master mix and 5 µl of primer mix (both 1.3 µM). Amplification was performed on an ABI 7500 Real-Time PCR system (ThermoFisher). Primer specificity was confirmed by analysis of the melting curves. Primer combinations had at least 90% efficiency. Relative transcript levels were calculated following the 2^–∆∆*C*t^ method (Livak and Schmittgen, 2001) using the geometric mean of the elongation factor (*EF-1A*), mitochondrial ATP-synthase beta subunit (*ATP2*), and ubiquitin (*UBQ*) as references. Primer amplification efficiency was determined using five standards from serial dilutions of a pool including the five time-points studied in this experiment.

### Preparation of the plant microsomal fraction and immunoblotting

A plant microsomal fraction was prepared and tested by immunoblotting as described previously (Bultreys *et al.*, 2009). The primary antibodies used were rabbit polyclonal antibodies against the whole plasma-membrane H^+^-ATPase family (Morsomme *et al.*, 1998), the plasma-membrane PIP aquaporins (Heinen *et al.*, 2014), the mitochondrial dihydrolipoyl dehydrogenase (DLD; de Castro Silva Filho *et al.*, 1996), or against NtPDR3. The anti-NtPDR3 antibody used in the first figure has been described previously (Ducos *et al.*, 2005), while that used in other figures was obtained as follows. The 120-bp DNA fragment encoding Pro-795 to Arg-834 of NtPDR3 was amplified by PCR using primers, one of which allowed the insertion of a six-histidine tag at the C-terminal end, and the PCR reaction product was inserted into the *Escherichia coli* expression vector pGEX-KG (Guan and Dixon, 1991). The glutathione S-transferase/NtPDR3 fusion protein was produced in *E. coli*, purified on a nickel–nitrilotriacetic acid agarose resin (Qiagen, Valencia, CA), and used to immunize rabbits.

### Plasma membrane enrichment

Plasma membranes were purified by partitioning in an aqueous two-phase system as described by Larsson *et al.* (1987).

### Chlorophyll quantification

Chlorophyll content was quantified according to Moran and Porath (1980).

### Transport assay on *N. tabacum* BY-2 cells

Cultures of 4-d-old *N. tabacum* BY-2 cells (200 ml) were incubated for 16 h at 37 °C. After 1 h of recovery at 25 °C, cell density was adjusted to 0.07 g cells ml^–1^ in MS medium and 5-ml aliquots were transferred to 6-well plates (Sterile 6-well Cell Culture Plate, Cellstar) and supplemented with 20 µM fraxetin. Cells were incubated in the dark for 60 min under agitation (90 rpm) at 25 °C. The cell suspensions were then filtered on 3MM cellulose filters. The extracellular media were collected in 10 ml Falcon tubes (Corning) and frozen in liquid nitrogen. The cells were directly transferred to tubes containing 600 mg of glass beads (0.8–1 mm diameter, VWR) and frozen in liquid nitrogen. All samples were stored at –20 °C until analysis.

Frozen samples of the extracellular medium were thawed on ice and aliquots of 400 µl were cleared using Ultrafree^®^-MC-GV centrifugal filters (Millipore). Cells were homogenized in 600 µl 85% (v/v) ULC-MS methanol supplemented with 0.1% formic acid three times for 40 s at 5500 rpm (Precellys 24, Bertin Technologies). The homogenate was cleared in the same way as for the extracellular medium samples, and fraxetin and its derivatives were quantified by HPLC-HRMS (Orbitrap) analysis. Separation of metabolites was carried out with an Accela 1250 HPLC system (ThermoFisher) equipped with a C_18_ Kinetex column (150 × 2.0 mm, internal diameter 3 µm; Phenomenex) using a linear gradient from 97% H_2_O (with 1% CH_3_CN and 0.1% HCOOH)/3% CH_3_CN to 40% H_2_O in 30 min. The flow rate was 0.2 ml min^–1^, and the column was maintained at 30 °C and the autosampler at 7 °C. Mass spectra were acquired with a Q-Exactive mass spectrometer (ThermoFisher Scientific) equipped with an electrospray ionization (ESI) source in the negative mode. ESI inlet conditions were as follows: spray voltage 3.2 kV, capillary temperature 290 °C, sheath gas 40 PSI, and auxiliary gas 3 PSI. The injection volume was 10 µl.

## Results

### NtPDR3 is a plasma-membrane protein expressed in plant roots in response to Fe deficiency

**Fig. 1.**
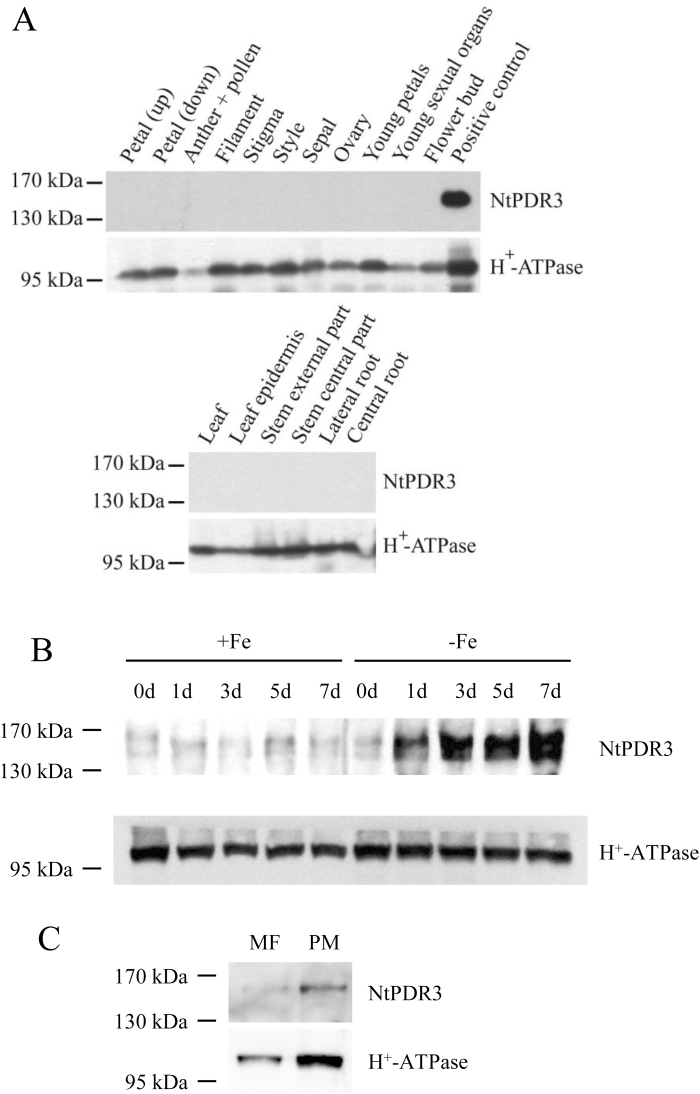
Organ-specific and subcellular expression of NtPDR3 and induction of *NtPDR3* expression by Fe deficiency in *N. tabacum* plants. (A) Microsomal fractions (20 µg) from different organs were tested by immunoblotting for NtPDR3, using H^*+*^-ATPase as the loading control. ‘Petal (up)’ and ‘Petal (down)’ correspond to the pink upper part and the white lower part, respectively, of a full-length petal. The positive control was a microsomal fraction of *N. tabacum* BY-2 cells incubated for 48 h in a medium supplemented with 1 mM Na_2_EDTA. (B) Root microsomal fractions (20 µg) prepared from plants grown under hydroponic conditions before transfer (0 d) and at 1, 5, and 7 d after transfer to medium with (+Fe) or without (–Fe) supplementation with 10 µM Fe(III)-EDTA, tested by immunoblotting for NtPDR3, using H^*+*^-ATPase as the loading control. (C) Microsomal fraction (MF) and plasma membrane-enriched fraction (PM) proteins (5 µg) from the roots of plants grown under hydroponic conditions in Fe-deficient medium for 3 d, tested by immunoblotting for NtPDR3, using H^*+*^-ATPase as a PM marker. Note that the protein concentration was reduced compared with (A, B) in order to better see the enrichment in the plasma-membrane fraction.

When *N. tabacum* plants were grown under normal conditions in agar and *NtPDR3* expression was assessed by immunoblotting in various organs using specific antibodies, no expression was detected in leaves, stems, roots, or reproductive organs (Fig. 1A).

Since *NtPDR3* expression was previously shown to be induced by Fe deficiency in *N. tabacum* BY-2 suspension cells (Ducos *et al.*, 2005), *N. tabacum* plants were grown hydroponically in the presence or absence of 10 μM Fe(III)-EDTA for up to 7 d and root microsomal fractions prepared after 1, 3, 5, or 7 d were tested by immunoblotting. Although very low signals were detected in fractions from Fe-fed plants, a band at about 160 kD, corresponding to the expected size of NtPDR3, increased over time in the fractions prepared from Fe-deficient plant roots (Fig. 1B). Iron shortage is usually accompanied by external acidification, with the plasma membrane H^+^-ATPase being suggested to play a role (Santi and Schmidt, 2009). However, western blotting showed that Fe shortage did not significantly modify the total amount of H^+^-ATPase (Fig 1B). This was confirmed using three different loading controls: Coomassie Blue-stained gel and western blotting (PIP and DLD) (Supplementary Fig. S2A). Activation of *PDR3* expression by Fe shortage was confirmed to occur at the transcript level using RT-qPCR (Supplementary Fig. S2B).

To determine the subcellular localization of NtPDR3, we used immunoblotting to compare the NtPDR3 signal in a microsomal fraction and in a phase partition-purified plasma-membrane fraction prepared from roots of *N. tabacum* plants grown under Fe-deficient conditions. As shown in Fig. 1C, the NtPDR3 signal enrichment in the plasma-membrane fraction was found to be similar to that of the H^+^-ATPase, a plasma-membrane marker, indicating that NtPDR3 localizes to the plant plasma membrane.

**Fig. 2.**
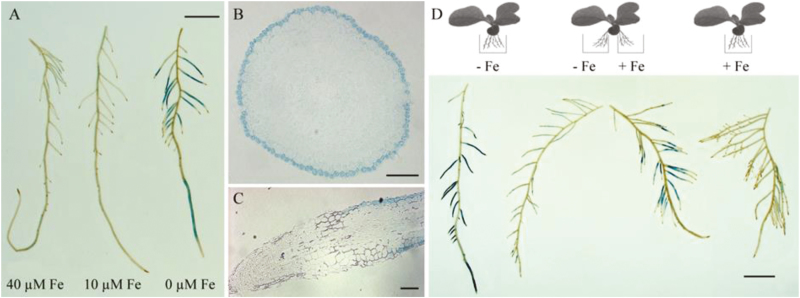
Tissue-specific localization of *NtPDR3* in *N. tabacum* plants. (A) GUS staining of *pNtPDR3::GUS* plant roots grown under hydroponic conditions 3 d after transfer to medium supplemented with 0, 10, or 40 µM Fe(III)-EDTA (scale bar =10 mm). (B) Cross-section and (C) longitudinal section of a Fe-deficient root (scale bars =0.1 mm). (D) *NtPDR3* expression in response to a localized Fe supply. The root system of a *pNtPDR3::GUS* plant grown under hydroponic conditions in the presence of Fe was split into two equal parts and one half was dipped in medium supplemented (+Fe) with 10 µM Fe(III)-EDTA and the other half in Fe-free medium (–Fe), while control plants had their whole root systems dipped in medium with or without Fe supplementation. GUS staining was then performed after 3 d of growth under these conditions (scale bar =10 mm). The results shown are representative of several independent *pNtPDR3::GUS* plant lines tested.

In order to further analyse *NtPDR3* expression, transgenic *N. tabacum* plants expressing the *GUS* gene fused to a 1502-bp fragment (Supplementary Fig. S3) of the putative *NtPDR3* transcription promoter (*pNtPDR3::GUS* plants) were raised and grown hydroponically in 0, 10, or 40 μM Fe(III)-EDTA for 3 d, and then GUS activity was assessed by histochemical staining. In the root, whereas *pNtPDR3::GUS* plants grown in Fe-supplemented medium showed only faint GUS activity, plants grown under Fe-depleted conditions displayed strong staining in the main and lateral roots (Fig. 2A). The staining was observed in the youngest part of the roots. Cross-sectioning showed that it was mainly restricted to the epidermal cell layer (Fig. 2B), whereas longitudinal sectioning revealed that it was absent in the meristematic zone of the root and appeared in a more basal region, probably the differentiation zone (Fig. 2C). No staining was observed in leaf tissues of *pNtPDR3::GUS* plants grown under Fe-deficient conditions (Supplementary Fig. S4).

Similar to previous observations for other genes induced in roots by Fe deficiency, such as *FRO2* and *IRT1* from Arabidopsis (Vert, *et al.*, 2002), *NtPDR3* induction by Fe deficiency was shown to be regulated by both local and long-distance signals. In a split-root experiment in which half of the *pNtPDR3::GUS* roots from a plant grown in a Fe-containing medium were dipped in Fe-containing medium and the other half in Fe-deficient medium, GUS staining was seen in both halves of the root, but was stronger in the part dipped in the Fe-containing medium (Fig. 2D). This expression pattern indicated that *NtPDR3* induction by Fe deficiency was, like that of *FRO2* or *IRT1*, subject to systemic positive regulation, probably through a shoot-borne signal, by the global Fe-deficient status of the plant and was also positively regulated by the presence of Fe in the medium at the root level.

### *NtPDR3*-silencing results in higher sensitivity to Fe deficiency

**Fig. 3.**
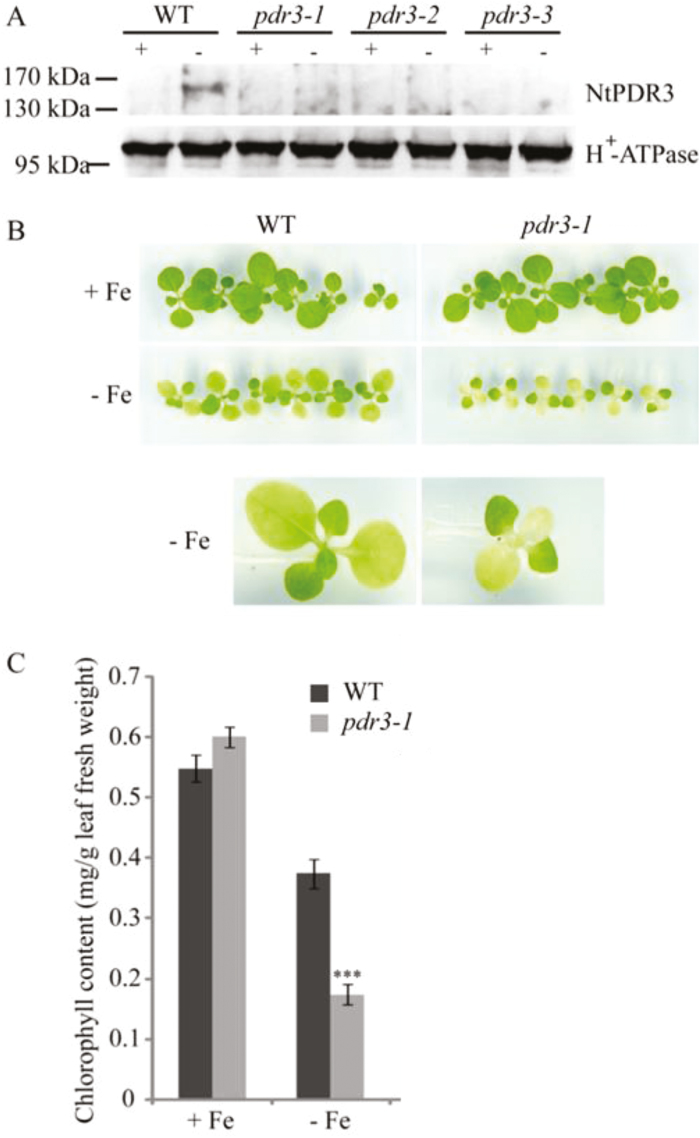
Phenotype of *N. tabacum* transgenic lines silenced for *NtPDR3* expression using artificial microRNA. (A) Root microsomal proteins (20 µg) were prepared from seedlings of three independent transgenic lines (*pdr3-1*, *pdr3-2*, and *pdr3-3*) and the wild-type (WT) grown under *in vitro* conditions for 12 d on medium with (+Fe) or without (–Fe) supplementation with 10 µM Fe(III)-EDTA, and tested by immunoblotting for NtPDR3, using H^*+*^-ATPase as the loading control. (B, C) Growth and chlorophyll content of the *pdr3-1* line compared to the wild-type (WT). (B) Images of 21-d-old plantlets grown under *in vitro* conditions for 13 d on medium with (+Fe) or without (–Fe) supplementation with 10 µM Fe(III)-EDTA. The images shown are representative of those from three independent experiments. At the bottom are enlarged images of a wild-type and a *pdr3-1* plantlet grown without Fe supplementation. Similar data were obtained for lines *pdr3-2* and *pdr3-3* (see Supplementary Fig. S5A). (C) Leaf chlorophyll content of the same plantlets. The data are means (±SE) of six measurements performed on individual seedlings. Significant differences compared with the wild-type were determined using Student’s *t*-test: ***P<0.001. Similar data were obtained for lines *pdr3-2* and *pdr3-3* (Supplementary Fig. S5B).

To better understand the role of *NtPDR3* in the plant response to Fe deficiency, we generated transgenic *N. tabacum* plants in which *NtPDR3* expression was prevented by artificial microRNA. Ten *NtPDR3*-silenced lines were identified among 15 transgenic lines and showed stable silencing. Three silenced lines (*pdr3-1*, *pdr3-2*, and *pdr3-3*) were used for more detailed characterization (Fig. 3A). These lines did not show any visible phenotypic change when grown in the presence of 10 µM Fe(III)-EDTA. However, when grown in Fe-depleted medium, *pdr3-1* (Fig. 3B) and *pdr3-2* and *pdr3-3* (Supplementary Fig. S5A) had smaller shoots than the wild-type and a stronger chlorotic leaf phenotype, with the youngest leaves turning white after 13 d of growth without Fe, in contrast to the wild-type leaves that remained light-green. When leaf chlorophyll was quantified, the content of the wild-type and the three mutant lines did not differ significantly under a normal Fe supply. However, when grown for 13 d on Fe-depleted medium, the mutants showed a decrease of ~60% in leaf chlorophyll content compared to the wild-type (Fig. 3C for *pdr3-1* and Supplementary Fig. S5B for *pdr3-2* and *pdr3-3*). These results showed that NtPDR3 is involved in the plant adaptation to Fe deficiency.

### NtPDR3 is involved in the export of O-methylated coumarins to the rhizosphere

In an attempt to identify NtPDR3 substrate(s), we collected the culture media of wild-type and *NtPDR3*-silenced plants grown hydroponically under both control and Fe-deficient conditions. We hypothesized that the secretion of such substrate(s) would be induced by Fe deficiency and would be more abundant in the wild-type exudates than in the mutant ones. Since AtPDR9, the Arabidopsis homolog of NtPDR3, has been found to be involved in the secretion of coumarins (Fourcroy *et al.*, 2014; Ziegler *et al.*, 2017), we used conditions favorable for its identification. From a total of approximately 200 possible mass spectral features analysed per run and per sample, only 13 compounds complied with the following criteria: (i) occurring at the retention time (RT) where absorbance at 320 nm was observed; (ii) showing peak area increases (or appearing) with Fe-deficiency; and (iii) not being ions associated with adducts. The RTs, exact values of *m/z*, assigned elemental formula, and MS^n^ fragmentations of these compounds, labelled E1–E13, are shown in Supplementary Table S2. Nine were identified as *O*-methylated coumarins and four as flavins.

**Fig. 4.**
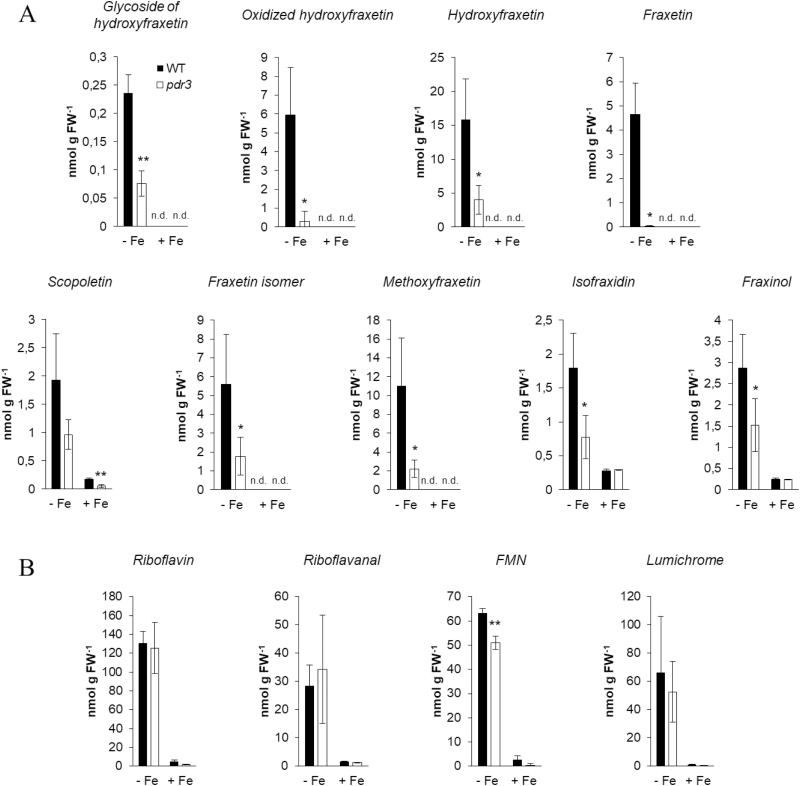
Effect of *NtPDR3*-silencing on root exudation of coumarins and flavins from *N. tabacum* plants. Coumarins (A) and flavins (B) were quantified in the nutrient solution of wild-type (WT) and *NtPDR3*-silenced (*pdr3*) plants grown under hydroponic conditions 3 d after their transfer to medium with (+Fe) or without (–Fe) supplementation with 10 µM Fe(III)-EDTA. Data are means (±SD) of three independent biological replicates. Significant differences compared with the wild-type were determined using Student’s *t*-test: *P<0.05; **P<0.01; ***P<0.001.

In order to investigate the involvement of NtPDR3 in the export of the identified metabolites to the nutrient solution, quantification of coumarins and flavins was carried out using [M+H]^+^ signals in HPLC/ESI-MS (TOF) chromatograms. Iron deficiency induced a massive export of both coumarins and flavins in the nutrient solution of *N. tabacum* 3 d after the onset of the treatment (Fig. 4). In total, flavins were almost six times more abundant than coumarins (287 nmol g^–1^ versus 49 nmol g^–1^ FW, respectively). Riboflavin was the most abundant flavin in the exudates (45% of the total flavins). The most abundant coumarin was hydroxyfraxetin (33%) followed by methoxyfraxetin (22%). Interestingly, a glycosylated form of hydroxyfraxetin was also identified in the exudates. However, its abundance was relatively low, accounting for less than 0.5% of the total coumarins.

*NtPDR3*-silencing caused a marked decrease in the secretion of all coumarins to the nutrient solution (Fig. 4A), whereas flavin secretion (Fig. 4B) was unaffected, with only the exception of a small decrease for FMN (Fig. 4B). When compared to their concentrations in wild-type exudates, the largest decrease was for fraxetin (193-fold), followed by oxidized hydroxyfraxetin (19-fold), methoxyfraxetin (5-fold), hydroxyfraxetin (4-fold), the isomer of fraxetin (3-fold), and scopoletin, isofraxidin, and fraxinol (2-fold each). The concentration of the only glycoside detected in the culture medium, the glycoside of hydroxyfraxetin, was also decreased 3-fold in the exudates of *NtPDR3*-silenced lines.

### *NtPDR3*-silenced roots accumulate more glycosylated coumarins than the wild-type

In order to confirm that NtPDR3 is involved in the secretion of coumarins, but not flavins, in response to Fe deficiency, methanolic extracts from roots were also obtained and analysed. We hypothesized that if a substrate of NtPDR3 was less abundant in the exudates of mutant plants, it should accumulate within the roots of such plants. As for the exudate samples, root extracts were analysed by reverse-phase C_18_ HPLC coupled to UV/Vis and both ESI-MS (TOF) and ESI-MS/MS (ion trap). We found 12 compounds complied with the same three criteria listed above, and details are shown in Supplementary Table S3, with the compounds labelled R1–R12. As expected, the 12 compounds absorbing at 320 nm and being increased by Fe-deficiency in *N. tabacum* roots were flavins and coumarins. The four flavins identified were the same as those already observed in the nutrient solutions. However, coumarins identified in the roots were mainly glycosides, instead of the aglycone coumarins found in the nutrient solutions.

**Fig. 5.**
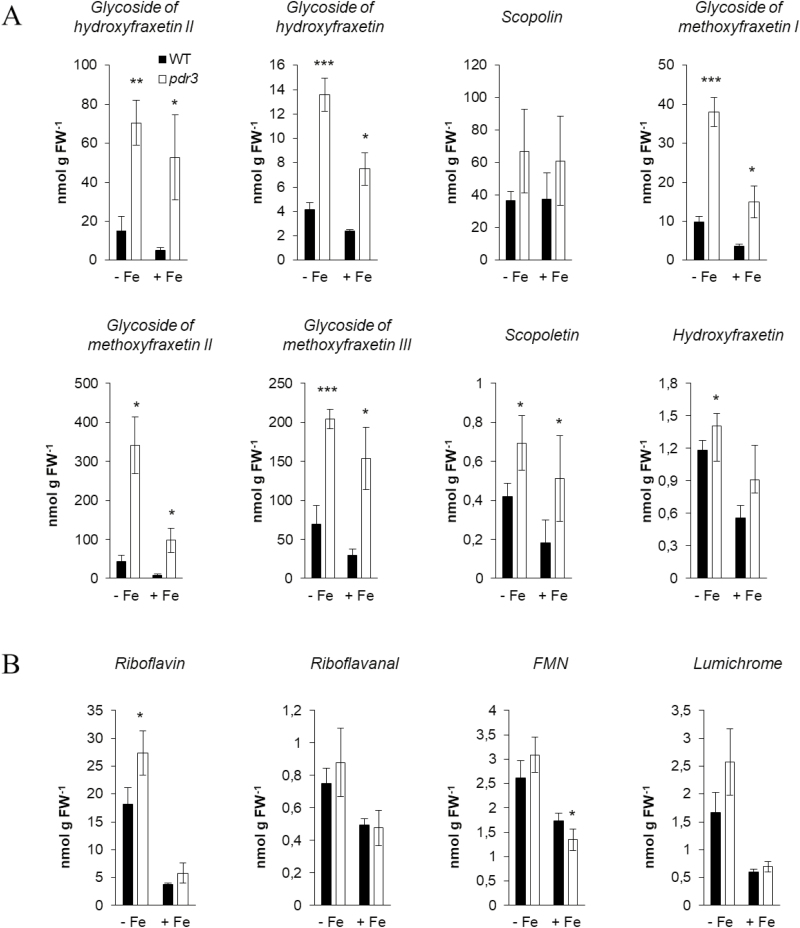
Effect of *NtPDR3*-silencing on root accumulation of coumarins and flavins from *N. tabacum* plants. Coumarins (A) and flavins (B) were quantified in roots extracts obtained from wild-type (WT) and *NtPDR3*-silenced (*pdr3*) plants grown under hydroponic conditions, 3 d after their transfer to medium with (+Fe) or without (–Fe) supplementation with 10 µM Fe(III)-EDTA. Data are means (±SD) of three independent biological replicates. Significant differences compared with the wild-type were determined using Student’s *t*-test: *P<0.05; **P<0.01; ***P<0.001.

The quantification of metabolites revealed that the roots of the *NtPDR3*-silenced lines accumulated more coumarins than the wild-type (Fig. 5A). When compared to their concentrations in wild-type roots, the largest increase was for the second isomer of methoxyfraxetin glucoside (8-fold), followed by the first isomer of hydroxyfraxetin glucoside (5-fold). As observed in the nutrient solutions, flavin concentrations did not seem to be markedly modified in roots of *NtPDR3*-silenced plants, except for a 50% increase in riboflavin (Fig. 5B).

### Expression of *NtPDR3* in *N. tabacum* BY-2 cells modulates the coumarin pools

**Fig. 6.**
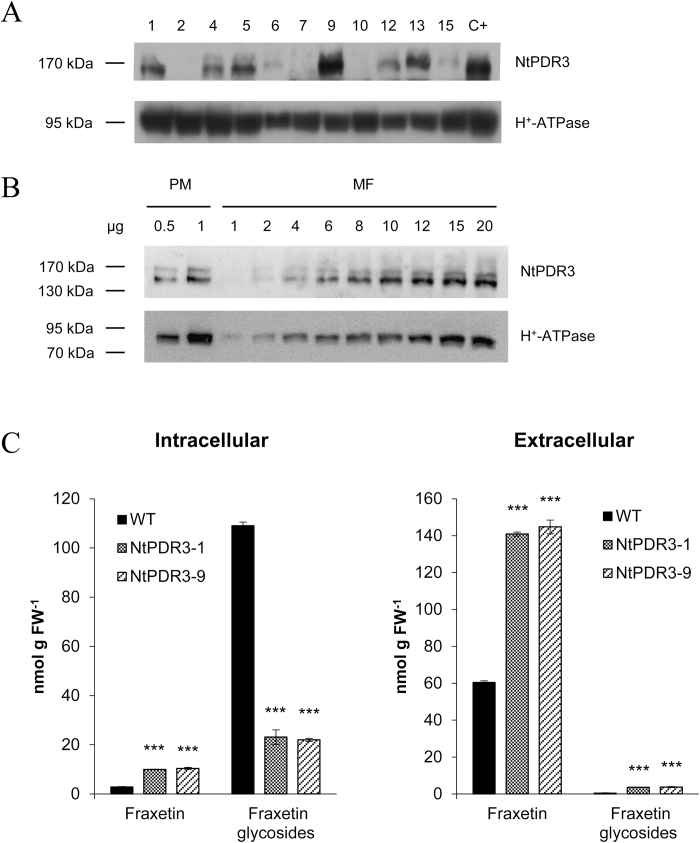
NtPDR3 expression modulates *N. tabacum* BY-2 intracellular and extracellular coumarin pools. (A) Overexpression of *NtPDR3* in different BY-2 suspension cell lines (numbered at the top). Microsomal fractions (20 µg) were prepared 16 h after induction at 37 °C and subjected to western blotting for detection of NtPDR3, using H^+^-ATPase as a loading control. Microsomal fractions prepared from wild-type BY-2 cells induced for NtPDR3 expression by a treatment of 48 h with 1 mM Na_2_EDTA were used as a positive control (C+). (B) Microsomal fraction (MF) and plasma membrane-enriched (PM) proteins from *N. tabacum* BY-2 cells overexpressing NtPDR3 (line NtPDR3-9). The weight of samples loaded is indicated (µg). (C) One wild-type (WT) and two *NtPDR3*-overexpressing cell lines (NtPDR3-1 and NtPDR3-9) were incubated in the presence of 20 µM fraxetin for 1 h. Fraxetin and its glycosides were quantified in cellular methanolic extracts (left) as well as in the extracellular culture medium (right). Data are means (±SE) of three biological replicates. Significant differences compared with the wild-type were determined using Student’s *t*-test: ***P<0.001.

The observation that *NtPDR3*-silenced plants secreted less *O*-methylated coumarins to the rhizosphere, whilst in parallel accumulating more of their glycosides in their roots, suggested that NtPDR3 transports these phenylpropanoids. To demonstrate this in a more direct manner, we sought to express NtPDR3 provided with a 10-His/STREPII/TEV tag in *N. tabacum* Bright Yellow-2 (BY-2) suspension cells using the constitutive En2pPMA4 promoter (De Muynck *et al.*, 2009). Upon *A. tumefaciens*-mediated transformation, no NtPDR3-expressing transformant was obtained despite this expression system having been successfully used for other PDR transporters (Pierman *et al.*, 2017; Toussaint *et al.*, 2017). This suggested that constitutive NtPDR3 expression is lethal. We therefore made use of the heat-induced transcription promoter *NtHSP3A* (Navarre *et al.*, 2011). Several transgenic lines showed *NtPDR3* expression after incubation at 37 °C for 16 h (Fig. 6A). In one of these lines (NtPDR3-9), we compared the NtPDR3 signal in a microsomal fraction and in a phase partition-purified plasma-membrane fraction (Fig. 6B). The signal was strongly enriched in the plasma-membrane fraction, but to a lesser extent than the marker for H^+^-ATPase, suggesting that NtPDR3 was partly in internal membranes.

We used fraxetin as a model coumarin for transport experiments as this compound is not produced *de novo* by BY-2 cells, is commercially available, and was found to display the largest decrease in the *NtPDR3*-silenced exudates in the metabolic profiling experiment (193-fold compared to the wild-type). Upon heat induction, wild-type and two *NtPDR3*-expressing cell lines (NtPDR3-1 and NtPDR3-9) were incubated in the presence of 20 µM fraxetin, and the intracellular and extracellular concentrations were quantified after 60 min. As fraxetin was found to be rapidly glycosylated by endogenous glycosyltransferases (Supplementary Fig. S6), these forms were also quantified.

When the extracellular medium of wild-type BY-2 cells was supplemented with fraxetin, 63% of the fraxetin molecules were glycosylated, and the vast majority of these glycosylated forms were found in the intracellular extract (Fig. 6C). However, the two *NtPDR3*-expressing lines were found to accumulate 5-fold less glycosylated coumarins intracellularly, and these differences were mainly compensated by a higher proportion of fraxetin detected in the extracellular medium. However, more fraxetin was also detected in the intracellular fractions of the *NtPDR3*-expressing lines. One additional interesting observation was that after incubation with fraxetin, both *NtPDR3*-expressing lines accumulated small amounts of glycosylated fraxetin in the extracellular medium (accounting for 2% of the total fraxetin pool) (Fig. 6C).

## Discussion

**Fig. 7.**
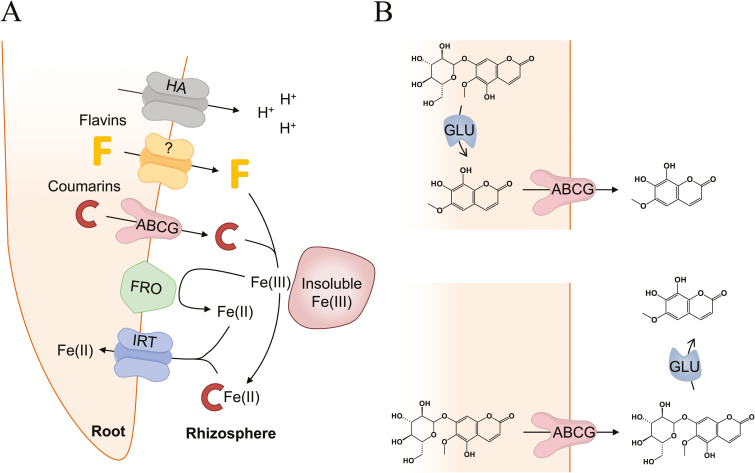
Schematic mechanism of Fe uptake by *N. tabacum* plants. (A) The reduction-based Fe uptake Strategy I used by all higher plants except for those of the Graminacae family relies mainly on the reduction of ferric chelates at the root surface through the activity of the ferric-chelate reductase FRO. The ferrous Fe thus generated is then taken up into root cells by the Fe-regulated transporter IRT. To increase the solubility of ferric Fe and to support the reducing capacity of Fe^3+^ at the root surface, these plants also activate P-type H^+^-ATPases (HA) to acidify the rhizosphere. In addition, Strategy I plants also secrete species-dependent secondary metabolites with Fe-chelating and/or -reducing properties. Whereas Arabidopsis secretes only coumarins (C) through a full-size ATP-binding cassette (ABCG) transporter, other species such as *Medicago truncatula* and *Beta vulgaris* secrete only flavins (F) through an unknown mechanism. In *N. tabacum* plants, both systems are present. Adapted from Sisó-Terraza *et al.* (2016*b*). Flavins secreted by roots of iron-deficient *Beta vulgaris* enable mining of ferric oxide via reductive mechanisms. New Phytologist 209, 733–745; and Tsai and Schmidt (2017). Mobilization of iron by plant-borne coumarins. Trends in Plant Science 22, 538–548, with permission from Elsevier. (B) The current model proposes that coumarins are deglycosylated by β -glucosidases (GLU) in the cytosol prior their export to the rhizosphere (upper panel). Our data support an alternative route, namely that coumarins are exported as glycosides and removal of the sugar moiety occurs in the apoplast (lower panel).

Iron homeostasis involves Fe uptake from the soil, its compartmentalization within the cell, and its distribution throughout the plant. In *N. tabacum*, the response to Fe deficiency includes classical Strategy I responses as well as the elicitation of root accumulation and secretion of both coumarins and flavins (Fig. 7A). The data from our study provide strong evidence that *NtPDR3* plays an important role in the plant’s adaptation to Fe deficiency. *NtPDR3* expression was detected at a low level in roots under normal conditions by RT-PCR, immunoblotting, and using the *GUS*-reporter gene, but it was clearly up-regulated in roots when Fe was absent (Fig. 1, Supplementary Fig. S2). Its expression was restricted to the epidermis (Fig. 2) and the protein was enriched in plasma-membrane preparations as compared to microsomal fractions (Fig. 1C). Interestingly, Fe shortage did not significantly modify the level of the plasma-membrane H^+^-ATPase. Although acidification of the external medium is a usual response to Fe deficiency, the role of H^+^-ATPase in this acidification has been questioned to some extent (Vansuyt *et al.*, 2003). AHA2, a major H^+^-ATPase isoform in Arabidopsis was shown to be more expressed in roots upon Fe starvation (Santi and Schmidt, 2009) but this was not confirmed in a more recent proteomic analysis (Lan *et al.*, 2011). The data presented here confirm the absence of activation of H^+^-ATPase expression in *N. tabacum*. This does not exclude activation of this enzyme at the activity level. Indeed, this enzyme is regulated by phosphorylation at several sites as well as by binding of 14-3-3 regulatory proteins (Duby and Boutry, 2009; Haruta *et al.*, 2015; Falhof *et al.*, 2016).

Taken together, the data for expression and subcellular localization point to a role of NtPDR3 in the plant response to Fe deficiency. An important indication of this came from the characterization of transgenic plants silenced for this transporter. These plants displayed growth defects and lower chlorophyll content on Fe-deficient medium compared to the wild-type (Fig. 3).

Comparing the root metabolites and exudates of wild-type and *NtPDR3*-silenced plants grown in hydroponics allowed us to identify alterations in pools of *O*-methylated coumarins (Figs 4, 5). Mutant roots accumulated more *O*-methylated coumarins, mainly in the form of glycosides, whilst at the same time they secreted less of these metabolites to the rhizosphere. These results are reminiscent of those obtained in an Arabidopsis knockout mutant of AtPDR9, the homolog of NtPDR3 (Rodríguez-Celma *et al.*, 2013; Fourcroy *et al.*, 2014; Ziegler *et al.*, 2017; Rajniak *et al.*, 2018; Tsai *et al.*, 2018). However, several differences can be noted between the two species. First, the set of secreted coumarins differ, since in *N. tabacum* 96% of the total consisted of highly-substituted coumarins including two tetra-oxygenated (44% hydroxyfraxetin, including its oxidized form, and 22% methoxyfraxetin) and three tri-oxygenated forms (9% fraxetin, 11% fraxetin isomer, 6% fraxinol, and 4% isofraxidin). The remaining 4% corresponded to the di-oxygenated coumarin scopoletin. Conversely, in Arabidopsis, according to initial reports, only 63–72% of the total corresponds to tri- and tetra-oxygenated coumarins already found in *N. tabacum*, with the exception of methoxyfraxetin, whereas 28–37% of the total corresponds to two di-oxygenated coumarins, scopoletin and esculetin (Sisó-Terraza *et al.*, 2016*a*; Ziegler *et al.*, 2017; Tsai *et al.*, 2018). However, the experimental set-up, and particularly the pH of the nutrient solution, seems to greatly impact the nature of the coumarins identified. Indeed, very recently, Rajniak *et al.* (2018) showed that hydroxyfraxetin (referred to as sideretin) was the major coumarin exuded by Arabidopsis in response to Fe deficiency, at least under acidic conditions, whereas alkaline conditions favored exudation of less-oxidized coumarins, such as scopoletin and fraxetin. A second fundamental discrepancy with Arabidopsis is that our metabolic approach also allowed us to determine that upon Fe deficiency, *N. tabacum* roots secreted a set of flavins (riboflavin, riboflavanal, FMN, and lumichrome) in addition to coumarins, and also that NtPDR3 did not seem to be involved in the exudation of these metabolites (Fig. 7). This observation is interesting as secretion of flavins and coumarins was previously thought to be mutually exclusive (Rodríguez-Celma *et al.*, 2013). In fact, Fe deficiency-induced secretion of riboflavin was previously reported to occur either alone (*Hysociamus albus*, *Petroselinum crsipum*) or in combination with other flavins (two riboflavin sulfates in *Amarathus caudatus*, *Beta vulgaris*, and *Spinacea oleracea*; three oxygenated-flavins in *M. truncatula*; riboflavanal in *Cucumis sativus*; and an unknown flavin in *C. melo*) (Rodríguez-Celma *et al.*, 2011; Hsieh and Waters, 2016; Satoh *et al.*, 2016). It should be stressed that coumarins and flavins can take part in functions other than Fe acquisition. For instance, they also function in deterring feeding by pests, allelopathic activity against plant competitors, responses to microbial pathogen attack, and in adaptive mechanisms to phosphorus limitation (Jordan *et al.*, 1992; Susín, 1994; Gnonlonfin *et al.*, 2012; Ziegler *et al.*, 2016; Stringlisa *et al.*, 2018).

Given that the available data for AtPDR9 and NtPDR3 mutant plants did not exclude the possibility that other transporters induced by Fe deficiency are involved in coumarin secretion, we took an additional step by following NtPDR3 transport activity in another cellular context and in the absence of Fe deficiency. Expression of the transporter in *N. tabacum* BY-2 suspension cells was relevant for several reasons. First, the background expression of *NtPDR3* was very low. Second, in this homologous system, NtPDR3 was correctly targeted to the plasma membrane. This was not the case in our attempts to express *NtPDR3* in *Saccharomyces cerevisiae*, where NtPDR3 was retained in internal membranes (data not shown). Interestingly, unlike for other *N. tabacum* PDR transporters (Toussaint *et al.*, 2017; Pierman *et al.*, 2017), constitutive NtPDR3 expression could not be obtained in *N. tabacum* BY-2 cells. This might indicate that NtPDR3 transports a metabolite essential for BY-2 cell survival. The heat-induced promoter turned out to be a useful tool, allowing strong expression upon induction. Fraxetin (7,8-dihydro-6-methoxycoumarin) was used as a model coumarin as it is not produced *de novo* by BY-2 cells, it is commercially available, and it was found to display the largest decrease in the *NtPDR3*-silenced exudates compared to the wild-type in our metabolic profiling experiment. Expression of NtPDR3 in BY-2 cells clearly confirmed coumarin transport (Fig. 6).

Our results indicated that coumarins are stored as glycosides within the roots of *N. tabacum*, as previously shown for Arabidopsis. This observation is also in line with what has been reported for many other phenylpropanoids (Hino *et al.*, 1982; Whetten and Sederoff, 1995; Alejandro *et al.*, 2012; Väisänen *et al.*, 2015). This is easily explained by the fact that aglycone forms of phenylpropanoids are highly nucleophilic and therefore unstable and putatively cytotoxic. The attachment of carbohydrate moieties reduces their reactivity, improves their stability, and can control compartmentalization and biological activity (Jones and Vogt, 2001; Le Roy *et al.*, 2016). In *N. tabacum*, a UDP-glucosyltransferase with high affinity towards hydroxycoumarins, NtTOGT1, has been shown to be involved in scopoletin glycosylation *in planta* (Chong *et al.*, 2002). Once glycosylated, most phenylpropanoids, and most likely coumarins, are stored in the vacuole (Dima *et al.*, 2015). However, no specific vacuolar transporter of glycosylated coumarins has yet been identified.

As our metabolic profiling experiment identified coumarins almost exclusively in their aglycone form in the exudates of Fe-deficient *N. tabacum* roots, it seems that β-glucosidase-mediated removal of sugar moieties occurs at some point, either before or after secretion (Fig. 7B). Some previous observations support the hypothesis that the removal of the sugar moiety of coumarin glycosides occurs before secretion. In Arabidopsis, four β-glucosidases have been shown to mediate hydrolysis of glycosylated coumarins. Three of these (BGLU21, BGLU22, and BGLU23/PYK10) are localized in endoplasmic reticulum (ER)-bodies and have been shown to specifically hydrolyse scopolin to its corresponding aglycone scopoletin *in vitro* (Ogasawara *et al.*, 2009; Ahn *et al.*, 2010). Interestingly, these three β-glucosidases seem to be stored in ER-bodies in an inactive form, and require binding to the cytosolic PYK10-binding protein 1 (PBP1) to be activated (Nagano *et al.*, 2005, 2008). On the other hand, BGLU42 is a cytosolic β-glucosidase that also appears to mediate hydrolysis of coumarin glycosides. Interestingly, *bglu42* mutants accumulate more and secrete less coumarins, suggesting that BGLU42 is required for the processing of coumarins before secretion. Moreover, BLGU42 is predominantly expressed in root hairs and is up-regulated upon Fe deficiency (Zamioudis *et al.*, 2014).

However, other observations tend to support removal of the sugar moiety of coumarin glycosides after secretion. Both the presence of hydroxyfraxetin glycoside in exudates of Fe-deficient wild-type *N. tabacum* roots and the occurrence of other uncharacterized β-glucosidases with predicted apoplastic localization (Xu *et al.*, 2004) could suggest that coumarin glycosides might constitute the transported compounds. The increased level of hydroxyfraxetin glycoside II in *ntpdr3* roots and their corresponding decrease in the exudates further support this hypothesis (Figs 4, 5). These data rule out the possibility that this compound had merely diffused through the roots into the nutrient solution. However, since no other coumarin glycoside was detected in the exudates of Fe-deficient plants, this would mean that the hexose moiety of hydroxyfraxetin glycoside II is removed less efficiently than that of other coumarins. Investigating this assumption would be an interesting way to further support the ‘transport prior to hydrolysis’ hypothesis. Another interesting observation was that NtPDR3 expression in *N. tabacum* BY-2 cells resulted in increased level of fraxetin within the cells. This might be related to the observation that NtPDR3 was not targeted to the plasma membrane with the same efficiency as the plasma-membrane marker H^+^-ATPase and that it was partly localized to internal membranes. In this case, glycosylated fraxetin might be partly transported in a compartment of the secretory pathway and further de-glycosylated by one of the ER-associated glucosidases mentioned above.

In conclusion, expression of *NtPDR3* in *N. tabacum* BY-2 cells modulates the intracellular and extracellular pools of fraxetin added to the medium. However, the fact that fraxetin is glycosylated in BY-2 cells, as is also the case *in planta*, makes the interpretation of our results more complex. Nonetheless, these results seem to provide additional experimental evidence that NtPDR3 (and AtPDR9 in Arabidopsis) could transport glycosylated coumarins rather than their corresponding aglycones (Fig. 7B). Indeed, our results show that cells expressing NtPDR3 have an increased efflux of fraxetin glucosides upon incubation with fraxetin than wild-type cells (Fig. 6C). Given the complexity of the *in vivo* situation, performing *in vitro* transport assays using purified NtPDR3 in reconstituted liposomes seems to be the only way to ultimately assess which coumarin form is actually transported by NtPDR3.

## Supplementary data

Supplementary data are available at *JXB* online.

Fig. S1. Amino acid sequence of the tag added at the N-terminus of the *NtPDR3* sequence.

Fig. S2. Induction of NtPDR3 expression in the absence of Fe.

Fig. S3. Nucleotide sequence of the *NtPDR3* transcription promoter region.

Fig. S4. NtPDR3 is specifically induced by Fe deficiency in roots.

Fig. S5. Phenotype of the *N. tabacum pdr3-2* and *pdr3-3* lines under Fe-deficiency conditions.

Fig. S6. HPLC-MS chromatogram of BY-2 cell extracts incubated in the presence of fraxetin.

Table S1. Primers used for the RT-qPCR analysis.

Table S2. Metabolites identified in the exudates of *N. tabacum* in response to Fe deficiency.

Table S3. Metabolites identified in the roots of *N. tabacum* in response to Fe deficiency.

Supplementary Figures S1-S6 and Tables S1-S3

## References

[CIT0001] Ahn YO, Shimizu B, Sakata K, Gantulga D, Zhou C, Zhou Z, Bevan DR, Esen A 2010 Scopolin-hydrolyzing beta-glucosidases in roots of Arabidopsis. Plant & Cell Physiology 51, 132–143. 19965874 10.1093/pcp/pcp174

[CIT0002] Alejandro S, Lee Y, Tohge T, et al 2012 AtABCG29 is a monolignol transporter involved in lignin biosynthesis. Current Biology 22, 1207–1212. 22704988 10.1016/j.cub.2012.04.064

[CIT0003] Ambler JE, Brown JC, Gauch HG 1971 Sites of iron reduction in soybean plants. Agronomy Journal 63, 95–97.

[CIT0004] Balk J, Pilon M 2011 Ancient and essential: the assembly of iron-sulfur clusters in plants. Trends in Plant Science 16, 218–226. 21257336 10.1016/j.tplants.2010.12.006

[CIT0005] Bultreys A, Trombik T, Drozak A, Boutry M 2009 *Nicotiana plumbaginifolia* plants silenced for the ATP-binding cassette transporter gene *NpPDR1* show increased susceptibility to a group of fungal and oomycete pathogens. Molecular Plant Pathology 10, 651–663. 19694955 10.1111/j.1364-3703.2009.00562.x PMC6640336

[CIT0006] Cesco S, Neumann G, Tomasi N, Pinton R, Weisskopf L 2010 Release of plant-borne flavonoids into the rhizosphere and their role in plant nutrition. Plant Soil 329, 1–25.

[CIT0007] Chong J, Baltz R, Schmitt C, Beffa R, Fritig B, Saindrenan P 2002 Downregulation of a pathogen-responsive tobacco UDP-Glc:phenylpropanoid glucosyltransferase reduces scopoletin glucoside accumulation, enhances oxidative stress, and weakens virus resistance. The Plant Cell 14, 1093–1107. 12034899 10.1105/tpc.010436 PMC150609

[CIT0008] Dakora FD, Phillips DA 2002 Root exudates as mediators of mineral acquisition in low-nutrient environments. Plant Soil 245, 35–47.

[CIT0009] de Castro Silva Filho M, Chaumont F, Leterme S, Boutry M 1996 Mitochondrial and chloroplast targeting sequences in tandem modify protein import specificity in plant organelles. Plant Molecular Biology 30, 769–780. 8624408 10.1007/BF00019010

[CIT0010] De Muynck B, Navarre C, Nizet Y, Stadlmann J, Boutry M 2009 Different subcellular localization and glycosylation for a functional antibody expressed in *Nicotiana tabacum* plants and suspension cells. Transgenic Research 18, 467–482. 19140023 10.1007/s11248-008-9240-1

[CIT0011] Dima O, Morreel K, Vanholme B, Kim H, Ralph J, Boerjan W 2015 Small glycosylated lignin oligomers are stored in Arabidopsis leaf vacuoles. The Plant Cell 27, 695–710. 25700483 10.1105/tpc.114.134643 PMC4558659

[CIT0012] Duby G, Boutry M 2009 The plant plasma membrane proton pump ATPase: a highly regulated P-type ATPase with multiple physiological roles. Pflügers Archiv 457, 645–655. 18228034 10.1007/s00424-008-0457-x

[CIT0013] Ducos E, Fraysse S, Boutry M 2005 NtPDR3, an iron-deficiency inducible ABC transporter in *Nicotiana tabacum*. FEBS Letters 579, 6791–6795. 16337204 10.1016/j.febslet.2005.11.014

[CIT0014] Falhof J, Pedersen JT, Fuglsang AT, Palmgren M 2016 Plasma membrane H^+^-ATPase regulation in the center of plant physiology. Molecular Plant 9, 323–337. 26584714 10.1016/j.molp.2015.11.002

[CIT0015] Fourcroy P, Sisó-Terraza P, Sudre D, et al 2014 Involvement of the ABCG37 transporter in secretion of scopoletin and derivatives by Arabidopsis roots in response to iron deficiency. New Phytologist 201, 155–167. 24015802 10.1111/nph.12471

[CIT0016] Goderis IJ, De Bolle MF, François IE, Wouters PF, Broekaert WF, Cammue BP 2002 A set of modular plant transformation vectors allowing flexible insertion of up to six expression units. Plant Molecular Biology 50, 17–27. 12139006 10.1023/a:1016052416053

[CIT0017] Gnonlonfin GJB, Sanni A, Brimer L 2012 Review scopoletin – a coumarin phytoalexin with medicinal properties. Critical Reviews in Plant Sciences 31, 47–56.

[CIT0018] Guan KL, Dixon JE 1991 Eukaryotic proteins expressed in *Escherichia coli*: an improved thrombin cleavage and purification procedure of fusion proteins with glutathione S-transferase. Analytical Biochemistry 192, 262–267. 1852137 10.1016/0003-2697(91)90534-z

[CIT0019] Hänsch R, Mendel RR 2009 Physiological functions of mineral micronutrients (Cu, Zn, Mn, Fe, Ni, Mo, B, Cl). Current Opinion in Plant Biology 12, 259–266. 19524482 10.1016/j.pbi.2009.05.006

[CIT0020] Haruta M, Gray WM, Sussman MR 2015 Regulation of the plasma membrane proton pump (H^+^-ATPase) by phosphorylation. Current Opinion in Plant Biology 28, 68–75. 26476298 10.1016/j.pbi.2015.09.005 PMC4679459

[CIT0021] Heinen RB, Bienert GP, Cohen D, Chevalier AS, Uehlein N, Hachez C, Kaldenhoff R, Le Thiec D, Chaumont F 2014 Expression and characterization of plasma membrane aquaporins in stomatal complexes of *Zea mays*. Plant Molecular Biology 86, 335–350. 25082269 10.1007/s11103-014-0232-7

[CIT0022] Henriques R, Jásik J, Klein M, Martinoia E, Feller U, Schell J, Pais MS, Koncz C 2002 Knock-out of Arabidopsis metal transporter gene *IRT1* results in iron deficiency accompanied by cell differentiation defects. Plant Molecular Biology 50, 587–597. 12374293 10.1023/a:1019942200164

[CIT0023] Hino F, Okazaki M, Miura Y 1982 Effect of 2,4-dichlorophenoxyacetic acid on glucosylation of scopoletin to scopolin in tobacco tissue culture. Plant Physiology 69, 810–813. 16662301 10.1104/pp.69.4.810 PMC426310

[CIT0024] Horsch R, Fry J, Hoffmann NL, Eichholtz D, Rogers SG, Fraley RT 1985 A simple and general method for transferring genes into plants. Science 227, 1229–1231. 17757866 10.1126/science.227.4691.1229

[CIT0025] Hsieh EJ, Waters BM 2016 Alkaline stress and iron deficiency regulate iron uptake and riboflavin synthesis gene expression differently in root and leaf tissue: implications for iron deficiency chlorosis. Journal of Experimental Botany 67, 5671–5685. 27605716 10.1093/jxb/erw328 PMC5066488

[CIT0026] Jones P, Vogt T 2001 Glycosyltransferases in secondary plant metabolism: tranquilizers and stimulant controllers. Planta 213, 164–174. 11469580 10.1007/s004250000492

[CIT0027] Jordan CM, Wakeman RJ, Devay JE 1992 Toxicity of free riboflavin and methionine-riboflavin solutions to *Phytophthora infestans* and the reduction of potato late blight disease. Canadian Journal of Microbiology 38, 1108–1111.

[CIT0028] Lan P, Li W, Wen TN, Shiau JY, Wu YC, Lin W, Schmidt W 2011 iTRAQ protein profile analysis of Arabidopsis roots reveals new aspects critical for iron homeostasis. Plant Physiology 155, 821–834. 21173025 10.1104/pp.110.169508 PMC3032469

[CIT0029] Larsson C, Widell S, Kjellbom P 1987 Preparation of high-purity plasma membranes. Methods in Enzymology 148, 558–568.

[CIT0030] Le Roy J, Huss B, Creach A, Hawkins S, Neutelings G 2016 Glycosylation is a major regulator of phenylpropanoid availability and biological activity in plants. Frontiers in Plant Science 7, 735. 27303427 10.3389/fpls.2016.00735 PMC4880792

[CIT0031] Livak KJ, Schmittgen TD 2001 Analysis of relative gene expression data using real-time quantitative PCR and the 2^−ΔΔ*C*T^ method. Methods 25, 402–408. 11846609 10.1006/meth.2001.1262

[CIT0032] Maliga P, Sz-Breznovits A, Márton L 1973 Streptomycin-resistant plants from callus culture of haploid tobacco. Nature: New Biology 244, 29–30. 4515911 10.1038/newbio244029a0

[CIT0033] Marschner H, Römheld V, Kissel M 1986 Different strategies in higher plants in mobilization and uptake of iron. Journal of Plant Nutriton 9, 695–713.

[CIT0034] Moran R, Porath D 1980 Chlorophyll determination in intact tissues using n,n-dimethylformamide. Plant Physiology 65, 478–479. 16661217 10.1104/pp.65.3.478 PMC440358

[CIT0035] Morsomme P, Dambly S, Maudoux O, Boutry M 1998 Single point mutations distributed in 10 soluble and membrane regions of the *Nicotiana plumbaginifolia* plasma membrane PMA2 H^+^-ATPase activate the enzyme and modify the structure of the C-terminal region. The Journal of Biological Chemistry 273, 34837–34842. 9857010 10.1074/jbc.273.52.34837

[CIT0036] Nagano AJ, Fukao Y, Fujiwara M, Nishimura M, Hara-Nishimura I 2008 Antagonistic jacalin-related lectins regulate the size of ER body-type beta-glucosidase complexes in *Arabidopsis thaliana*. Plant & Cell Physiology 49, 969–980. 18467340 10.1093/pcp/pcn075

[CIT0037] Nagano AJ, Matsushima R, Hara-Nishimura I 2005 Activation of an ER-body-localized beta-glucosidase via a cytosolic binding partner in damaged tissues of *Arabidopsis thaliana*. Plant & Cell Physiology 46, 1140–1148. 15919674 10.1093/pcp/pci126

[CIT0038] Navarre C, Sallets A, Gauthy E, et al 2011 Isolation of heat shock-induced *Nicotiana tabacum* transcription promoters and their potential as a tool for plant research and biotechnology. Transgenic Research 20, 799–810. 21052831 10.1007/s11248-010-9459-5

[CIT0039] Ogasawara K, Yamada K, Christeller JT, Kondo M, Hatsugai N, Hara-Nishimura I, Nishimura M 2009 Constitutive and inducible ER bodies of *Arabidopsis thaliana* accumulate distinct beta-glucosidases. Plant & Cell Physiology 50, 480–488. 19147648 10.1093/pcp/pcp007

[CIT0040] Olsen RA, Bennett JH, Blume D, Brown JC 1981 Chemical aspects of the Fe stress response mechanism in tomatoes. Journal of Plant Nutrition 3, 905–921.

[CIT0041] Ossowski S, Schwab R, Weigel D 2008 Gene silencing in plants using artificial microRNAs and other small RNAs. The Plant Journal 53, 674–690. 18269576 10.1111/j.1365-313X.2007.03328.x

[CIT0042] Pierman B, Toussaint F, Bertin A, Lévy D, Smargiasso N, De Pauw E, Boutry M 2017 Activity of the purified plant ABC transporter NtPDR1 is stimulated by diterpenes and sesquiterpenes involved in constitutive and induced defenses. The Journal of Biological Chemistry 292, 19491–19502. 28972149 10.1074/jbc.M117.811935 PMC5702685

[CIT0043] Piette AS, Derua R, Waelkens E, Boutry M, Duby G 2011 A phosphorylation in the C-terminal auto-inhibitory domain of the plant plasma membrane H^+^-ATPase activates the enzyme with no requirement for regulatory 14-3-3 proteins. The Journal of Biological Chemistry 286, 18474–18482. 21482822 10.1074/jbc.M110.211953 PMC3099664

[CIT0044] Rajniak J, Giehl RFH, Chang E, Murgia I, von Wirén N, Sattely ES 2018 Biosynthesis of redox-active metabolites in response to iron deficiency in plants. Nature Chemical Biology 14, 442–450. 29581584 10.1038/s41589-018-0019-2 PMC6693505

[CIT0045] Robinson NJ, Procter CM, Connolly EL, Guerinot ML 1999 A ferric-chelate reductase for iron uptake from soils. Nature 397, 694–697. 10067892 10.1038/17800

[CIT0046] Rodríguez-Celma J, Lin WD, Fu GM, Abadía J, López-Millán AF, Schmidt W 2013 Mutually exclusive alterations in secondary metabolism are critical for the uptake of insoluble iron compounds by Arabidopsis and *Medicago truncatula*. Plant Physiology 162, 1473–1485. 23735511 10.1104/pp.113.220426 PMC3707556

[CIT0047] Rodríguez-Celma J, Vázquez-Reina S, Orduna J, Abadía A, Abadía J, Álvarez-Fernández A, López-Millán AF 2011 Characterization of flavins in roots of Fe-deficient strategy I plants, with a focus on *Medicago truncatula*. Plant & Cell Physiology 52, 2173–2189. 22039102 10.1093/pcp/pcr149

[CIT0048] Römheld V, Marschner H 1983 Mechanism of iron uptake by peanut plants. I. Fe^III^ reduction, chelate splitting, and release of phenolics. Plant Physiology 71, 949–954. 16662934 10.1104/pp.71.4.949 PMC1066149

[CIT0049] Santi S, Schmidt W 2009 Dissecting iron deficiency-induced proton extrusion in Arabidopsis roots. New Phytologist 183, 1072–1084. 19549134 10.1111/j.1469-8137.2009.02908.x

[CIT0050] Satoh J, Koshino H, Sekino K, et al 2016 *Cucumis sativus* secretes 4′-ketoriboflavin under iron-deficient conditions. Bioscience, Biotechnology, and Biochemistry 80, 363–367. 10.1080/09168451.2015.1095070 26523955

[CIT0051] Schmid NB, Giehl RF, Döll S, Mock HP, Strehmel N, Scheel D, Kong X, Hider RC, von Wirén N 2014 Feruloyl-CoA 6′-Hydroxylase1-dependent coumarins mediate iron acquisition from alkaline substrates in Arabidopsis. Plant Physiology 164, 160–172. 24246380 10.1104/pp.113.228544 PMC3875798

[CIT0052] Schmidt H, Günther C, Weber M, Spörlein C, Loscher S, Böttcher C, Schobert R, Clemens S 2014 Metabolome analysis of *Arabidopsis thaliana* roots identifies a key metabolic pathway for iron acquisition. PLoS ONE 9, e102444. 25058345 10.1371/journal.pone.0102444 PMC4109925

[CIT0053] Sisó-Terraza P, Luis-Villarroya A, Fourcroy P, Briat JF, Abadía A, Gaymard F, Abadía J, Álvarez-Fernández A 2016a Accumulation and secretion of coumarinolignans and other coumarins in *Arabidopsis thaliana* roots in response to iron deficiency at high pH. Frontiers in Plant Science 7, 1711. 27933069 10.3389/fpls.2016.01711 PMC5120119

[CIT0054] Sisó-Terraza P, Rios JJ, Abadía J, Abadía A, Álvarez-Fernández A 2016b Flavins secreted by roots of iron-deficient *Beta vulgaris* enable mining of ferric oxide via reductive mechanisms. New Phytologist 209, 733–745. 26351005 10.1111/nph.13633

[CIT0055] Stringlisa IA, Yua K, Feussnerb K, et al 2018 MYB72-dependent coumarin exudation shapes root microbiome assembly to promote plant health. Proceedings of the National Academy of Sciences, USA 115, E5213–E5222. 10.1073/pnas.1722335115 PMC5984513 29686086

[CIT0056] Susín S 1994 Respuestas inducidas por la deficiencia de hierro en el sistema radicular de Beta vulgaris L. PhD Thesis. Zaragoza University, Spain.

[CIT0057] Toussaint F, Pierman B, Bertin A, Lévy D, Boutry M 2017 Purification and biochemical characterization of NpABCG5/NpPDR5, a plant pleiotropic drug resistance transporter expressed in *Nicotiana tabacum* BY-2 suspension cells. The Biochemical Journal 474, 1689–1703. 28298475 10.1042/BCJ20170108

[CIT0058] Tsai HH, Rodríguez-Celma J, Lan P, Wu YC, Vélez-Bermúdez IC, Schmidt W 2018 Scopoletin 8-hydroxylase-mediated fraxetin production is crucial for iron mobilization. Plant Physiology 177, 194–207. 29559590 10.1104/pp.18.00178 PMC5933141

[CIT0059] Tsai HH, Schmidt W 2017 Mobilization of iron by plant-borne coumarins. Trends in Plant Science 22, 538–548. 28385337 10.1016/j.tplants.2017.03.008

[CIT0060] Väisänen EE, Smeds AI, Fagerstedt KV, Teeri TH, Willför SM, Kärkönen A 2015 Coniferyl alcohol hinders the growth of tobacco BY-2 cells and *Nicotiana benthamiana* seedlings. Planta 242, 747–760. 26108783 10.1007/s00425-015-2348-7

[CIT0061] van der Fits L, Deakin EA, Hoge JH, Memelink J 2000 The ternary transformation system: constitutive virG on a compatible plasmid dramatically increases *Agrobacterium*-mediated plant transformation. Plant Molecular Biology 43, 495–502. 11052201 10.1023/a:1006440221718

[CIT0062] Vansuyt G, Souche G, Straczek A, Briat JF, Jaillard B 2003 Flux of protons released by wild type and ferritin over-expressor tobacco plants: effect of phosphorus and iron nutrition. Plant Physiology and Biochemistry 41, 27–33.

[CIT0063] Varotto C, Maiwald D, Pesaresi P, Jahns P, Salamini F, Leister D 2002 The metal ion transporter IRT1 is necessary for iron homeostasis and efficient photosynthesis in *Arabidopsis thaliana*. The Plant Journal 31, 589–599. 12207649 10.1046/j.1365-313x.2002.01381.x

[CIT0064] Vert G, Grotz N, Dédaldéchamp F, Gaymard F, Guerinot ML, Briat JF, Curie C 2002 IRT1, an Arabidopsis transporter essential for iron uptake from the soil and for plant growth. The Plant Cell 14, 1223–1233. 12084823 10.1105/tpc.001388 PMC150776

[CIT0065] Whetten R, Sederoff R 1995 Lignin biosynthesis. The Plant Cell 7, 1001–1013. 12242395 10.1105/tpc.7.7.1001 PMC160901

[CIT0066] Xu Z, Escamilla-Treviño L, Zeng L, et al 2004 Functional genomic analysis of *Arabidopsis thaliana* glycoside hydrolase family 1. Plant Molecular Biology 55, 343–367. 15604686 10.1007/s11103-004-0790-1

[CIT0067] Yi Y, Guerinot ML 1996 Genetic evidence that induction of root Fe(III) chelate reductase activity is necessary for iron uptake under iron deficiency. The Plant Journal 10, 835–844. 8953245 10.1046/j.1365-313x.1996.10050835.x

[CIT0068] Zamioudis C, Hanson J, Pieterse CM 2014 β-Glucosidase BGLU42 is a MYB72-dependent key regulator of rhizobacteria-induced systemic resistance and modulates iron deficiency responses in Arabidopsis roots. New Phytologist 204, 368–379. 25138267 10.1111/nph.12980

[CIT0069] Ziegler J, Schmidt S, Chutia R, Müller J, Böttcher C, Strehmel N, Scheel D, Abel S 2016 Non-targeted profiling of semi-polar metabolites in Arabidopsis root exudates uncovers a role for coumarin secretion and lignification during the local response to phosphate limitation. Journal of Experimental Botany 67, 1421–1432. 26685189 10.1093/jxb/erv539 PMC4762384

[CIT0070] Ziegler J, Schmidt S, Strehmel N, Scheel D, Abel S 2017 Arabidopsis transporter ABCG37/PDR9 contributes primarily highly oxygenated coumarins to root exudation. Scientific Reports 7, 3704. 28623273 10.1038/s41598-017-03250-6 PMC5473935

