# Supplementary material for: The Nicotiana tabacum ABC transporter NtPDR3 secretes O-methylated coumarins in response to iron deficiency
Source: J Exp Bot. 2018 Jun 8;69(18):4419–31. doi: 10.1093/jxb/ery221 (PMC6093371; doi:10.1093/jxb/ery221)
Supplement: Supplementary Figures S1-S6 and Tables S1-S3 [file ery221_suppl_supplementary_figures_tables.pdf]

Supplementary Material

The *Nicotiana tabacum* ABC transporter NtPDR3 secretes O-methylated coumarins in response to iron deficiency

François Lefèvre[1‡*], Justine Fourmeau[1*], Mathieu Pottier[1], Amandine Baijot[1], Thomas Cornet[1], Javier Abadía[2], Ana Álvarez-Fernández[2] and Marc Boutry[1]

[1]Institut des Sciences de la Vie, Université catholique de Louvain, Croix du Sud, 4-5, Box L7-04-14, B-1348 Louvain-la-Neuve, Belgium
[2]Department of Plant Nutrition, Estación Experimental de Aula Dei, Consejo Superior de Investigaciones Científicas (CSIC), Apdo. 13034, E-50080 Zaragoza, Spain

*** These authors contributed equally to this work**

Figure S1. Amino acid sequence of the tag added at the N-terminus of the NtPDR3 sequence.

Affinity chromatography tags 10-His and StrepII are depicted in blue and green, respectively. The tobacco etch virus (TEV) protease cleavage site is in red and the N-terminus of NtPDR3 is in orange.

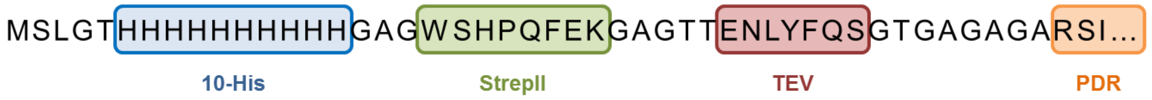

Figure S2. Induction of NtPDR3 expression in the absence of iron.

Root material was collected from *N. tabacum* plants grown under hydroponic conditions before transfer and one, three, five, or seven days (d) after transfer to medium without Fe and frozen at -80°C.

(A) A microsomal fraction was prepared and analyzed either by immunoblotting (10 µg) for NtPDR3, H+-ATPase DLD and PIP (upper panel) or by SDS-PAGE (30 µg) and Coomassie blue staining (lower panel).

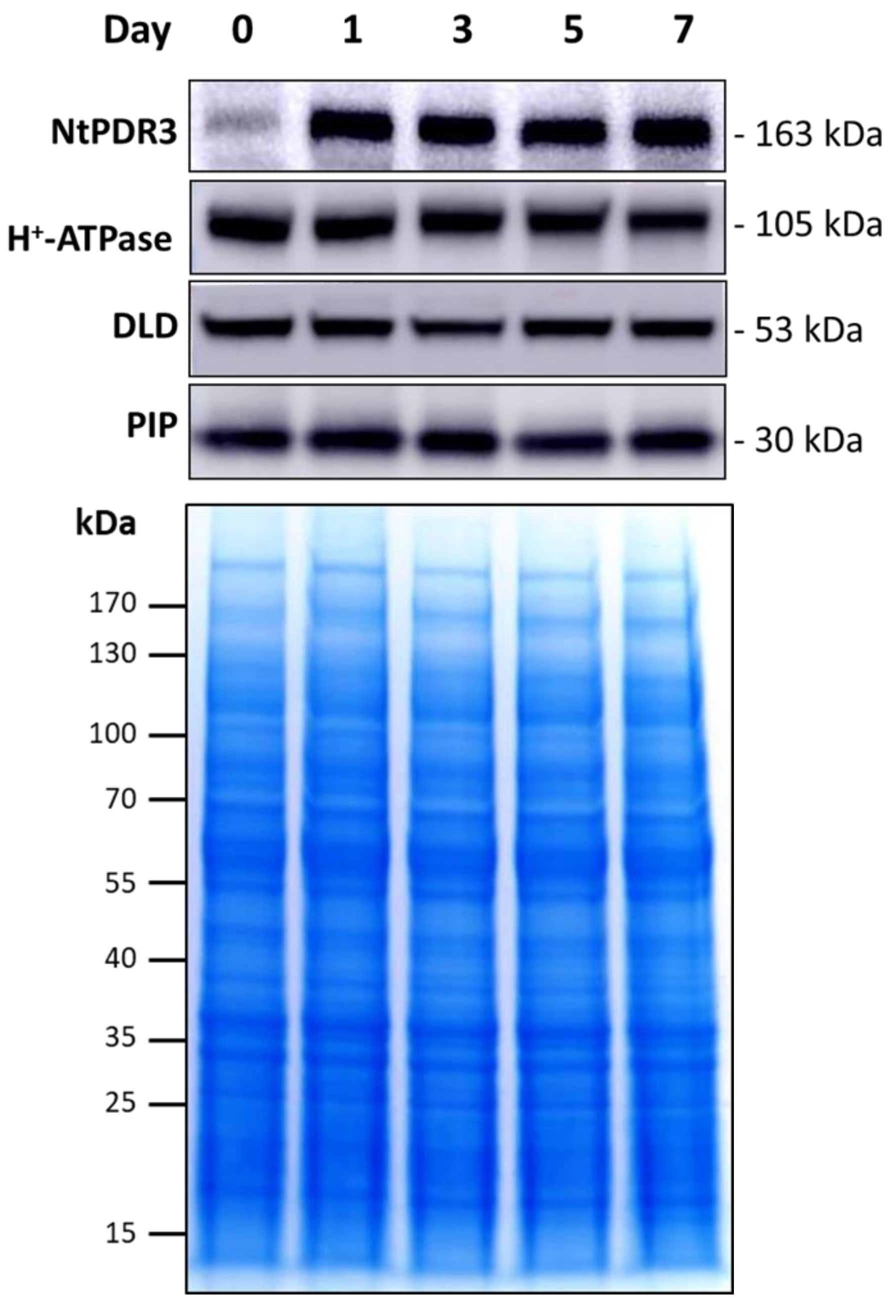

(B) Transcripts were prepared and quantified by RT-qPCR. The *NtPDR3* transcript level (set to 1) was normalized by the geometric mean of *NtEF-1α*, *NtATP2,* and *NtUBQ.* Results are shown as mean ± SE of four to eight repeats. Different letters indicate significant differences according to a Kruskal-Wallis test ($p < 0.05$) followed by a Tukey *post hoc* test.

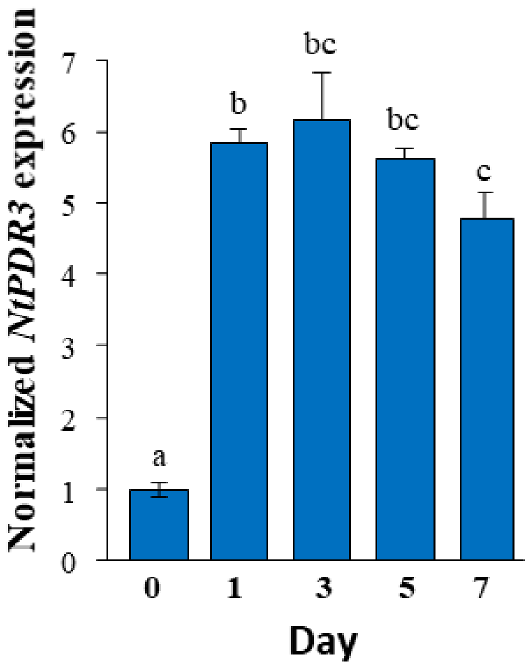

Figure S3. Nucleotide sequence of the *NtPDR3* transcription promoter region.

The nucleotide sequence of the *NtPDR3* transcription promoter region is displayed. The last three nucleotides (underlined) correspond to the translation initiation codon.

CGATTTGATTGGCTATGGAACTATCAGAAATCTTCTACCCACAAGGGTTAGGGCTGCCATGTGAGTATCAAGCCA
AAGTTTTGAGCTTTTTGCTGCTATTAAAATATTTCTGTCCACGTCATTGGGTCTAATTTATGTTCTGGGAATTTC
AATTAATATATGTTTCGGGAATTTATGTTGTTGTGATATTAATATTGTGTTCCTTTTTGTCCTTTTGTCTTTTTA
TTTTTTTGAGCCGAGGGTCTTTCGAAAATAGTCTCTTCAAAAATAGTCTATCTACTCCTTCGGGTAGGGGTAAAG
TCTGCGTACACATTACCCTCCCAGACCCTATTAGTGTGATTTTACTGGGTTGCTGTTGTTGTTGTTGTTGTTGCT
GTATGTTCCGGGAATTTAGTTACAAGTGAAAAACCAGGGAGAAGAAGGTTAGATTTTTTCTTGGTCCGATGTTAA
ACATTATTTTGTCTCCGCTAAGACCAATGATTTGGAAAAAGAAATTGCAAACTTAAAGGACTTGTAATGTTCAGT
AACAAATTTGAAGGACTAAAATAGTCCAACCCATAAACTTATGGGACCATTTGAGCCATTCTTTATTCTAGCTCT
AGACATGTAGGTTGATGAATAATTATGATATTTAGGTACGTAACCCATCCTTAATTTGACGTAAAACATAGCATT
ATTTAGTATTGGAATGAATTGGACCAATAGAGCTAGCAAATACTAATGATTAATTATGCAGATGGCAATTGATCA
GGCCTTACTTAGATTATGTAGTAGTTGGCTGTTCTTTCACTGATAAATGTTCATTTGATTGATACTAATATAGTT
TTGCTGTGTTGTAGTACTGTAATATTCATCTTCCTGGGGACCTTCTATTTAGCTGGGATGTCTCACGCCTTTACA
AGAGCTGACATACAGAACTCACCCTCCCCCACCCACCTCCCGCCAACTACAAAGAATAGTTGGAATATTATAATA
CATGCCATAAACAAAGCAGTAACGGGCATGGACCCAGCTATAATGGAAGCTCTATTGTAGATAAAGGAAGACCCT
AGCTCCTAGTTTAAGTCTTTGGAACTTTGGGAACTTATGGAACTTACATGTCAGAAACCTTAGAATAATCATTTC
TTTTTTGTCCTCCTAGGAAAGTCAACTGACTCTGTCCCTTGAGGAGTTAGAGAAATGTGCAGCACTCTTTTGTTT
ATTCAAAGATTTTTCTTTTGTTATTCTCCATAAACAAGCCTTATTTCATTTATTATCAACTGTAAACATCTGTTT
ACTCTCTAGGTATGTCTCTTTAATTTACGTTATCTATATAAAGAA*ATCAAGAATGCTTTTA*TTCATAAAAAAAAT
GAATGATGTATTTCCTTACCTCATCTATGTTACGGTCTAAATTTAGCTGTTCAAGCAAGCAAGAATATTTTATGT
TAGACATGCACGGTGCACCCCTCTTTGTTTTCTCCCTATAAATACAGCTTTGGAGCGGAAAATATTTTACACCAA
AA**ATG**

Figure S4. NtPDR3 is specifically induced by iron deficiency in roots. GUS staining of pNtPDR3::GUS *N. tabacum* grown for 7 days in a Fe-free medium. **(A)** Small developing leaf. **(B)** Section of a medium-size leaf. **(C)** section of a fully developed leaf**. (D)** Root. Scale bar = 1 cm.

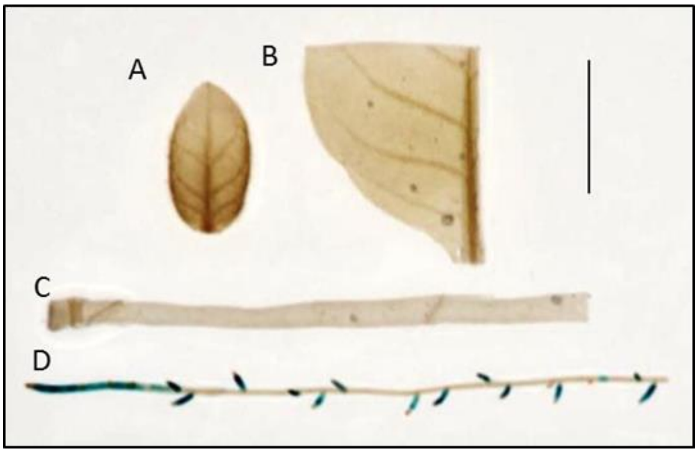

Figure S5. Phenotype of the *N. tabacum pdr3-2* and *pdr3-3* lines under iron deficiency conditions.

The growth and chlorophyll content of the *pdr3-2* and *pdr3-3* lines were compared to those for the wild-type (WT).

(A) Pictures of 21-day-old plantlets grown under *in vitro* conditions for 13 days on medium with (+Fe) or without (-Fe) supplementation with 10 µM Fe(III)-EDTA, 10 µM p-coumaric acid (p-CA), or 10 µM caffeic acid (CA). All media contained 0.1% methanol. The results shown are representative of those for three independent experiments.

(B) Leaf chlorophyll content of the same plantlets. The values are the mean ± SE for six measurements performed on individual seedlings. The asterisks indicate values significantly different from those for the wild-type grown under the same conditions (Student's test) (*, $P<0.05$; **, $P<0.01$; ***, $P<0.001$).

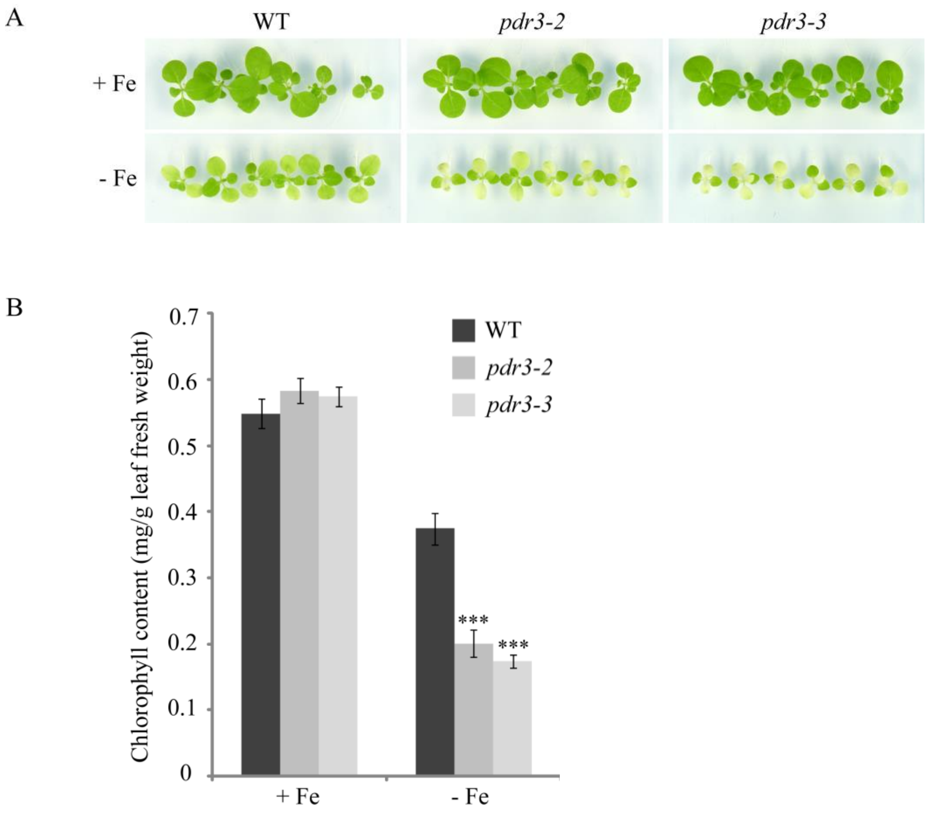

Figure S6. HPLC-MS chromatogram of BY-2 cell extracts incubated in the presence of fraxetin, extracted at *m/z* 369.08.

Typical HPLC/ESI-HRMS(Orbitrap) negative ionization mode chromatogram of the *m/z* 369.08 ion (corresponding to fraxetin hexosides) from a methanolic extract of BY-2 cells incubated with 20 µM fraxetin for 60 s.

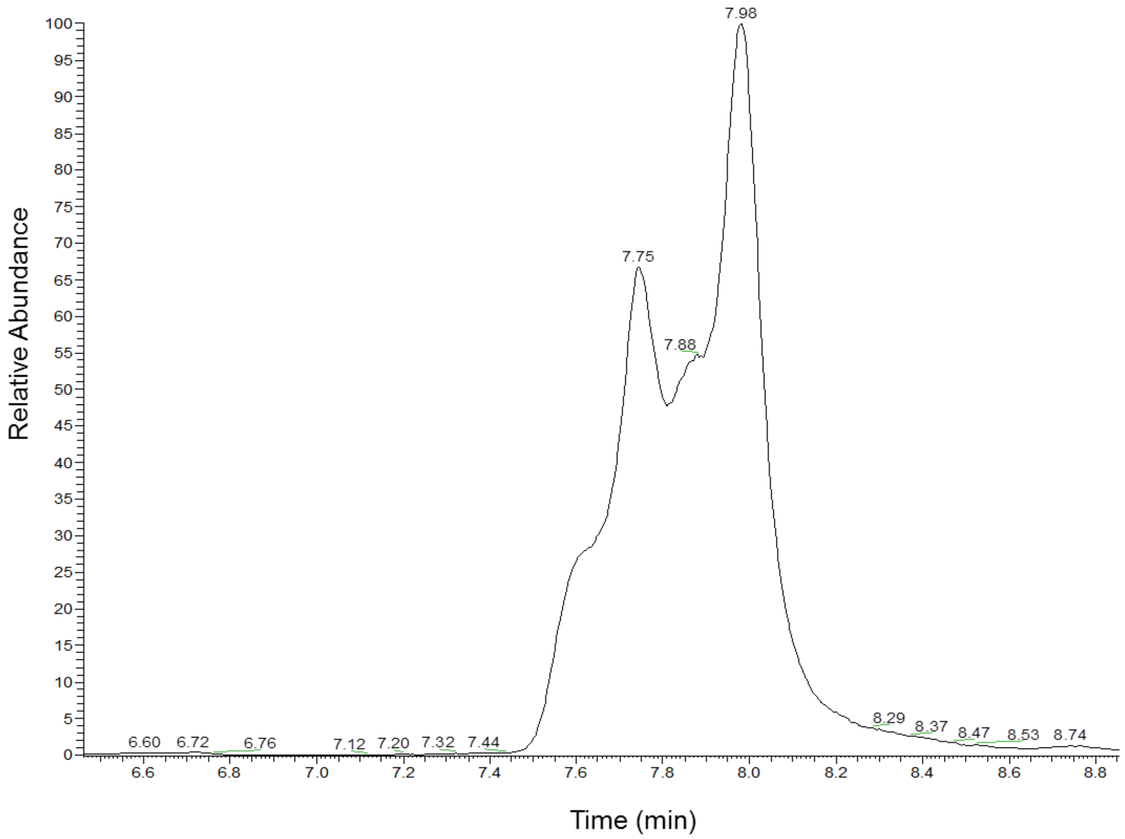

Table S1. Primers used for the RT-qPCR analysis.

Primers	Sequences (5’-3’)
qNtPDR3F	CAGCCTTTCTTGGGGACTATT
qNtPDR3R	CAACTTCCCAATGAAAAATGC
qNtATP2F	GGTTCCTTAGCCAGCCTTTC
qNtATP2R	CCAACACTCCCTGGAAACTG
qNtEF1aF	GGACATGCGTCAAACTGTTG
qNtEF1aR	TTCTTCTGAGCAGCCTTGGT
qNtUBQF	GAGGAATGCAGATCTTCGTG
qNtUBQR	TCCTTGTCCTGGATCTTAGC

Table S2. Metabolites identified in the exudates of *Nicotiana tabacum* in response to Fe deficiency.

Retention times (RT), exact mass-to-charge ratios (*m/z*), molecular formulae and error *m/z* (ppm). The *m/z* ratios of parent and fragment ions were determined from the HPLC/ESI-MS(TOF) and HPLC/ESI-MS(ion trap) chromatograms, respectively, working in positive mode.
[a] Identification based on standard. [b] Identification predicted on the literature (Fourcroy et al., 2014; Sisó-Terraza et al., 2016a,b; Satoh et al., 2016; Ziegler et al., 2017). Positions of the substitutions in the compound have not been confirmed by NMR studies.

#	R.T. (min)	Measured *m/z*	Molecular formula	Calculated *m/z*	Error *m/z* (ppm)	Annotation	ESI-MS^n *m/z* (Relative intensity %)
E1	7.6	387.0908	$C_{16}H_{19}O_{11}^+$	387.09219	-3.6	Hydroxyfraxetin glycoside (5,7,8-trihydroxy-6-methoxycoumarin hexoside)[b]	MS^2 [387]: 225 (100), 210 (5), 197 (1), 182 (1), 165 (1), 143 (1)<br>MS^3 [387→225]: 210 (100), 197 (3), 165 (9)
E2	11.8	223.0239	$C_{10}H_7O_6^+$	223.02371	-0.9	Oxidized hydroxyfraxetin (5-hydroxy-6-methoxy-2*H*-chromene-2,7,8-trione)[b]	MS^2 [223]: 205 (6), 195 (68), 177 (100), 167 (33), 149 (31), 139 (14), 121 (3)<br>MS^3 [223→195]: 185 (7), 180 (14), 167 (100), 139 (22)<br>MS^3 [223→177]: 149 (100), 121 (17)
E3	13.5	225.0395	$C_{10}H_9O_6^+$	225.03936	-0.6	Hydroxyfraxetin (5,7,8-trihydroxy-6-methoxycoumarin)[b]	MS^2 [225]: 210 (100), 197 (4), 179 (4), 165 (14), 151 (1), 119 (2), 100 (2)<br>MS^3 [225→210]: 191 (2), 182 (100), 164 (8), 154 (38), 136 (7), 126 (7)
E4	16.6	209.0442	$C_{10}H_9O_5^+$	209.04445	1.2	Fraxetin (7,8-dihydroxy-6-methoxycoumarin)[a]	MS^2 [209]: 194 (24), 181 (25), 177 (7), 163 (70), 153 (11), 149 (100), 135 (16), 107 (13)
E5	19.1	377.1453	$C_{17}H_{21}O_6N_4^+$	377.14556	0.7	Riboflavin (7,8-dimethyl-10-(2,3,4,5-tetrahydroxypentyl)flavin)[a]	MS^2 [377]: 359 (15), 243 (100), 216 (4), 172 (12)<br>MS^3 [377→243]: 216 (44), 202 (16), 190 (3), 172 (100)

E6	20.2	375.1295	$C_{17}H_{19}O_6N_4^+$	375.12991	1.1	Riboflavanal (7,8-dimethyl-10-(2,3,4-trihydroxypentanal)flavin)[b]	MS^2 [375]: 357 (100), 339 (17), 297 (4), 285 (15), 243 (50)<br>MS^3 [375→357]: 339 (5), 297 (16), 243 (100), 216 (5), 172 (9)<br>MS^3 [375)→243]: 216 (41), 200 (16), 190 (3), 172 (100)
E7	20.4	193.0493	$C_{10}H_9O_4^+$	193.04954	1.2	Scopoletin (7-hydroxy-6-methoxycoumarin)[a]	MS^2 [193]: 178 (11), 165 (22), 149 (13), 133 (100)
E8	21.0	209.0451	$C_{10}H_9O_5^+$	209.04445	3.6	Fraxetin isomer (5,7-dihydroxy-6-methoxycoumarin)[a]	MS^2 [209]: 197 (3), 194 (35), 187 (8), 186 (8), 185 (16), 182 (100), 181 (17)
E9	21.5	239.0546	$C_{11}H_{11}O_6^+$	239.05502	1.7	Methoxyfraxetin (7,8-dihydroxy-5,6-dimethoxycoumarin)[b]	MS^2 [239]: 224 (100), 209 (2), 206 (64), 179 (6)<br>MS^3 [239→224]: 209 (60), 206 (100), 196 (3), 178 (8)
E10	21.7	223.0599	$C_{11}H_{11}O_5^+$	223.0601	0.9	Isofraxidin (7-hydroxy-6,8-dimethoxycoumarin)[a]	MS^2 [223]: 208 (100), 195 (12), 190 (34), 179 (15), 163 (89), 145 (6), 135 (30), 119 (6), 107 (54)
E11	24.3	223.0598	$C_{11}H_{11}O_5^+$	223.0601	1.3	Fraxinol (6-hydroxy-5,7-dimethoxycoumarin)[a]	MS^2 [223]: 208 (100), 195 (9), 190 (54), 179 (7), 163 (56), 135 (13), 119 (2), 107 (2)
E12	27.5	457.1112	$C_{19}H_{17}O_8N_6P^+$	457.11189	-1.5	Flavin mononucleotide (FMN) (7,8-dimethyl-10-(2,3,4-trihydroxypentyl dihydrogen phosphate)flavin)[a]	MS^2 [457]: 439 (100), 421 (7), 396 (2), 359 (4), 341 (1), 243 (1)<br>MS^3 [457→439]: 421 (100), 403 (9), 396 (20), 359 (39), 341 (20), 323 (9), 297 (4), 243 (5), 172 (1)<br>MS^3 [457→359]: 341 (6), 316 (82), 298 (42), 288 (8), 281 (4), 254 (6), 243 (100), 226 (33), 216 (13), 210 (8), 200 (11), 172 (9), 116 (7)
E13	34.2	243.0878	$C_{12}H_{11}O_2N_4^+$	243.08765	-0.6	Lumichrome (7,8-dimethylflavin)[a]	MS^2 [243]: 216 (45), 200 (13), 190 (3), 172 (100)

Table S3. Metabolites identified in the roots of *Nicotiana tabacum* in response to Fe deficiency.

Retention times (RT), exact mass-to-charge ratios (*m/z*), molecular formulae and error *m/z* (ppm). The *m/z* ratios of parent and fragment ions were determined from the HPLC/ESI-MS(TOF) and HPLC/ESI-MS(ion trap) chromatograms, respectively, working in positive mode.
[a] Identification based on standard. [b] Identification predicted on the literature (Fourcroy et al., 2014; Sisó-Terraza et al., 2016a,b; Satoh et al., 2016; Ziegler et al., 2017). Positions of the substitutions in the compound have not been confirmed by NMR studies. When compounds were also present in nutrient solutions, labels indicated in Supplemental Table 1 are included in parentheses.

#	R.T. (min)	Measured *m/z*	Molecular formula	Calculated *m/z*	Error *m/z* (ppm)	Annotation	ESI-MS^n *m/z* (Relative intensity %)
R1	5.7	387.0905	$C_{16}H_{19}O_{11}^+$	387.09219	-4.4	Hydroxyfraxetin glycoside II (5,7,8-trihydroxy-6-methoxycoumarin hexoside)[b]	MS^2 [387]: 225 (100), 210 (8), 197 (1), 182 (1), 157 (1)<br>MS^3 [387→225]: 210 (100), 197 (2), 179 (2), 165 (10), 135 (1)
R2 (E1)	7.8	387.0910	$C_{16}H_{19}O_{11}^+$	387.09219	-3.1	Hydroxyfraxetin glycoside (5,7,8-trihydroxy-6-methoxycoumarin hexoside)[b]	/
R3	9.9	355.1021	$C_{16}H_{19}O_9^+$	355.10236	0.7	Scopolin (7-hydroxy-6-methoxycoumarin hexoside)[a]	MS^2 [355]: 232 (3), 193 (100), 133 (8)<br>MS^3 [355→193]: 178 (9), 165 (16), 161 (3), 149 (14), 137 (8), 133 (100)
R4	11.7	401.1093	$C_{17}H_{21}O_{11}^+$	401.10784	-3.6	Methoxyfraxetin glycoside I (7,8-dihydroxy-5,6-dimethoxycoumarin hexoside)[b]	MS^2 [401]: 239 (100), 224 (3), 206 (3), 178 (1)
R5	12.4	401.1086	$C_{17}H_{21}O_{11}^+$	401.10784	-1.9	Methoxyfraxetin glycoside II (7,8-dihydroxy-5,6-dimethoxycoumarin hexoside)[b]	MS^2 [401]: 239 (100), 224 (2), 206 (5), 195 (1)<br>MS^3 [301→239]: 224 (100), 206 (57), 179 (5), 157 (4), 137 (1)

R6	12,6	225.0387	$C_{10}H_9O_6^+$	225.03936	-2.9	Hydroxyfraxetin (5,7,8-trihydroxy-6-methoxycoumarin)[b]	MS^2 [225]: 207 (100), 193 (1), 175 (8), 147 (1)<br>MS^3 [225→207]: 192 (7), 175 (100), 147 (13), 119 (13)
R7	12.9	401.1091	$C_{17}H_{21}O_{11}^+$	401.10784	-3.1	Methoxyfraxetin glycoside III (7,8-dihydroxy-5,6-dimethoxycoumarin hexoside)[b]	MS^2 [401]: 239 (100), 224 (4), 206 (3), 179 (1)
R8 (E5)	19.0	377.1450	$C_{17}H_{21}O_6N_4^+$	377.14556	1.5	Riboflavin (7,8-dimethyl-10-(2,3,4,5-tetrahydroxypentyl)flavin)[a]	MS^2 [377]: 359 (15), 243 (100), 216 (4), 172 (11)<br>MS^3 [377→243]: 216 (45), 202 (13), 190 (4), 172 (100)
R9 (E6)	20.0	375.1297	$C_{17}H_{19}O_6N_4^+$	375.12991	0.6	Riboflavanal (7,8-dimethyl-10-(2,3,4-trihydroxypentanal)flavin)[b]	MS^2 [375]: 357 (100), 339 (15), 297 (3), 285 (15), 243 (48)<br>MS^3 [375)→243]: 216 (42), 200 (13), 190 (3), 172 (100)
R10 (E7)	20.3	193.0489	$C_{10}H_9O_4^+$	193.04954	-3.3	Scopoletin (7-hydroxy-6-methoxycoumarin)[a]	MS^2 [193]: 178 (13), 165 (25), 149 (10), 133 (100)
R11 (E12)	26.8	457.1098	$C_{19}H_{17}O_8N_6P^+$	457.11189	-4.6	Flavin mononucleotide (FMN) (7,8-dimethyl-10-(2,3,4-trihydroxypentyl dihydrogen phosphate)flavin)[a]	MS^2 [457]: 439 (100), 421 (5), 414 (1), 396 (1), 359 (6), 341 (1), 323 (1), 243 (1)<br>MS^3 [457→439]: 421 (100), 403 (13), 396 (32), 378 (3), 359 (55), 341 (37), 334 (4), 323 (14), 297 (10), 243 (17), 172 (2)
R12 (E13)	34.1	243.0874	$C_{12}H_{11}O_2N_4^+$	243.08765	1.0	Lumichrome (7,8-dimethylflavin)[a]	MS^2 [243]: 216 (43), 200 (14), 190 (4), 172 (100), 145 (3)
